# Sulfur-Based Ylides in Transition-Metal-Catalysed Processes

**DOI:** 10.1007/s41061-018-0193-4

**Published:** 2018-04-13

**Authors:** James D. Neuhaus, Rik Oost, Jérémy Merad, Nuno Maulide

**Affiliations:** Institute of Organic Chemistry, University of Vienna, Währinger Straße 38, 1090 Vienna, Austria

**Keywords:** Sulfonium ylides, Sulfoxonium ylides, Transition metal catalysis, Recent developments, Historical perspective, One-carbon synthon, Asymmetric catalysis

## Abstract

Traditionally employed in the synthesis of small ring systems and rearrangement chemistry, sulfur-based ylides occupy a unique position in the toolbox of the synthetic organic chemist. In recent years a number of pioneering researchers have looked to expand the application of these unorthodox reagents through the use of transition metal catalysis. The strength and flexibility of such a combination have been shown to be of key importance in developing powerful novel methodologies. This chapter summarises recent developments in transition metal-catalysed sulfonium/sulfoxonium ylide reactions, as well as providing a historical perspective. In overviewing the successes in this area, the authors hope to encourage others into this growing field.

## Introduction

Sulfur-based ylides are zwitterionic compounds in which each formal charge is stabilised by its proximity to the other. They have long been used as one-carbon synthons in a number of classical transformations, most notably for the synthesis of small rings such as epoxides, aziridines and cyclopropanes through ylide addition to electron-poor π-systems. While reactions with unstabilised sulfonium and sulfoxonium ylides are known, most modern processes utilise stabilised versions, in which the negative charge is delocalised into one or more electron withdrawing groups. This added stability means that sulfonium/sulfoxonium ylides can be used as practical, bench-stable reagents and enables the development of reactions of increased complexity. However, this extra stability is accompanied by a drop in the reactivity of the processes mentioned above. By combining sulfur ylides with transition metal catalysis, the utility of these unusual reagents can be greatly enhanced. In this chapter we seek to summarise the recent developments in this field. We have subdivided the reactions into three sections: (1) carbene-free processes; (2) reactions that employ sulfonium or sulfoxonium ylides as carbene precursors; (3) reactions that form sulfonium ylides in situ from metal carbenoids for cascade reactions. These distinctions were recently used in a related review, currently in press, in which the authors of the current review described such reactions in the context of structure and bonding [1].

## Metal–Ylide Complexes

Most successful transition metal-catalysed reactions involving sulfonium ylides rely on activation of either the sulfonium ylide or the coupling partner by the transition metal complex. In order to study and develop these potential processes, it is often instructive to investigate the coordination chemistry that would be invoked. To this end, a number of publications detailing metal–ylide complexes have been published over recent decades.

Initial studies in 1974 began with investigations on Pd(II) salts [2] and Pt(II) salts [2, 3]. In these studies, X-ray crystallographic analysis showed that for carbonyl-stabilised ylides, *σ*-coordination through the ylide carbon-atom was the preferred mode of bonding, rather than the alternative oxygen-bound possibility. A similar ambidentate character has been observed for a number of ylides and is well summarised in a 1983 review by Weber [4].

In the intervening years, carbon-based coordination chemistry of sulfonium ylides with alternative metal complexes has been reported for Pd(II) [1], Pt(II) [2, 3], Hg(II) [5], Cd(II) [6], and Ag(I) [7], while for hard, oxophilic metals such as W(0) co-ordination through oxygen has been observed [8].

**Scheme 1:**
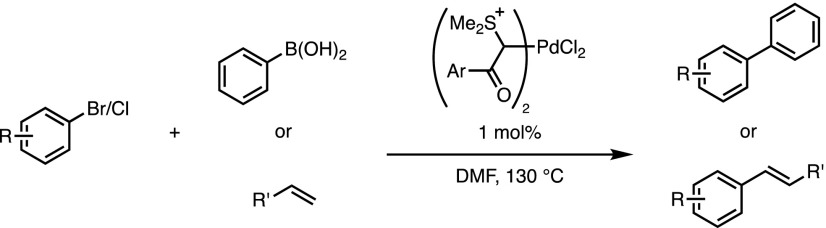
Sulfonium ylides complexes as cross-coupling catalysts.* DMF* Dimethylformamide

Beyond the simple study of the coordination chemistry, there are a number of publications detailing the use of sulfonium ylides as ligands in Pd(II)-catalysed processes. Suzuki–Miyaura reactions between aryl bromides or chlorides and aryl boronic acids have been promoted efficiently, with reported turnover numbers as high as 1940, and catalyst loadings as low as 0.05 mol% have been employed [9–11] (Scheme 1). Mizoroki–Heck reactions of aryl halides can also be promoted by the same catalyst [12]. Relative to alternative phosphine-free conditions, stability to aerobic conditions and the efficiency compare very favourably, although there remains scope to optimise and expand this concept.

## Non-Carbene-Based Transition Metal-Mediated Reactions of Sulfur Ylides

### Sulfur Ylides as Single-Carbon Synthons in Formal (*n *+ 1) Cycloadditions

**Scheme 2:**
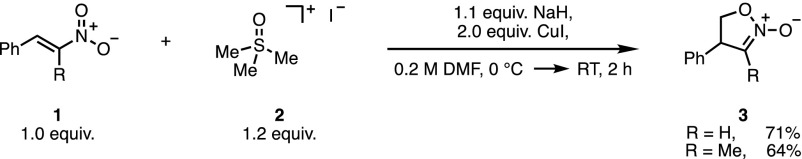
The first Cu-promoted formal (4 + 1) cycloaddition

The ability of sulfur-based ylides to efficiently participate in transition metal-catalysed (*n *+ 1) cycloadditions was fortuitously discovered by the group of Holy in the mid-1970s [13]. In order to achieve the cyclopropanation of β-nitrostyrenes (**1**) the authors explored a Corey–Chaykovsky-type process involving the ylide generated from sulfoxonium iodide **2**. The deprotonation of this salt with sodium hydride in the presence of nitro olefins **1** resulted in the immediate formation of an unidentified amorphous precipitate. In order to stabilise the sulfoxonium ylide intermediate, a stoichiometric amount of copper iodide was added. Under such modified conditions, compound **1** readily reacted with the preformed copper ylide complex while the unidentified precipitate was not observed. The major product was unexpectedly identified as a five-membered 2-isoxazoline *N*-oxide **3** (Scheme 2). The rationalisation of why the 5-*exo*-tet, *O*-based ring closure is favoured over the 3-*exo*-tet, *C*-based cyclisation is not trivial. Indeed, additional experiments demonstrated a close relationship between the nature of the Michael acceptor and the reaction outcome; the use of cinnamonitrile and diethyl benzylidenemalonate resulted in polymerisation, while α,β-unsaturated ketones and esters afforded a mixture of cyclopropane and recovered starting material.

**Fig. 1:**
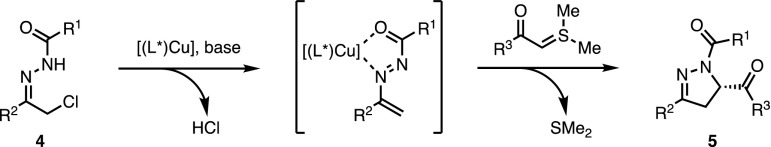
Chiral Lewis acid-promoted, formal (4 + 1) cycloaddition

**Scheme 3:**
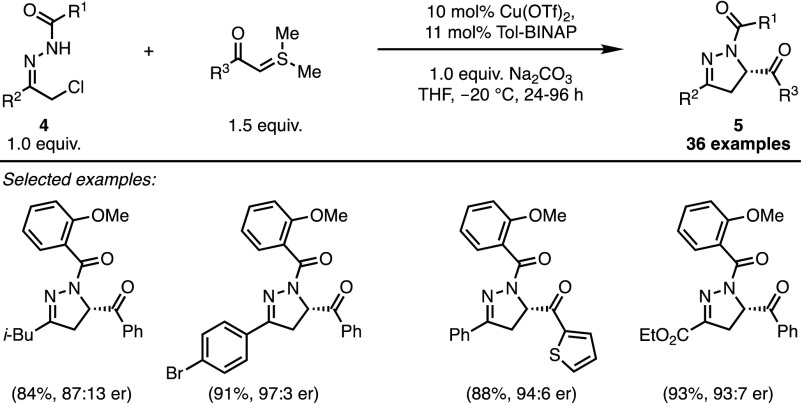
Scope of dihydropyrazole formation

Despite the synthetic potential offered by these observations, the reaction suffered from considerable drawbacks, such as a narrow scope and the need for stoichiometric amounts of metal salts to tame the high reactivity of the unstabilised sulfoxonium ylide. For these reasons, the metal-triggered formal (4 + 1) cycloaddition of sulfoxonium ylides did not garner any significant interest for almost 40 years. Recent developments in Lewis acid and hydrogen bonding-catalysed activation of 1,4-dipoles, however, opened new opportunities in this field [14]. In 2012, Bolm and co-workers revisited the formal (4 + 1) cycloaddition of sulfonium ylides by designing a remarkable catalytic and enantioselective version of this transformation [15]. In this case, a highly reactive azoalkene was generated, in situ, from the deprotonation of α-halo hydrazones **4** by Na_2_CO_3_ (Fig. 1). Such compounds had previously been shown to be extremely competent Michael-acceptors [16]. Subsequent enantioselective addition of a stabilised sulfonium ylide to this intermediate was promoted by a chiral BINAP–copper complex and resulted in the formation of enantioenriched dihydropyrazoles **5** (Scheme 3). Interestingly, stabilised ketosulfonium ylides proved to be the best substrates for this reaction. While for many classical ylide transformations these compounds are usually considered to be somewhat unreactive, this precise property renders them easier to control in metal-mediated processes. In particular, their poor Lewis basicity prevents irreversible co-ordination to the copper complex and allows catalytic turnover. In practice, the process displayed considerable robustness and remained efficient when performed on a gram scale. By demonstrating the synthetic potential of this strategy to access optically active five-membered heterocycles, Bolm precipitated a renewed interest for the use of sulfonium ylides as carbene synthons in catalytic cycloadditions.

**Scheme 4:**
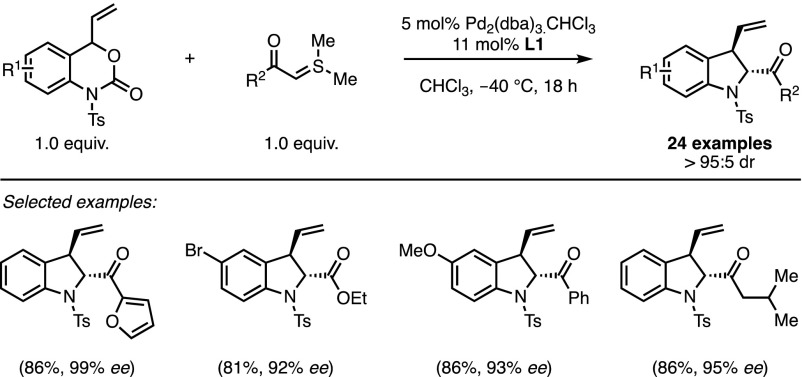
Palladium-catalysed decarboxylative (4 + 1) for the asymmetric synthesis of indolines

In the following years, major developments in this field involved the generation of 1,4-dipoles promoted by transition metal catalysts from readily available substrates. In this framework, stabilised sulfonium ylides have displayed a remarkable tolerance toward a range of catalytic systems. In 2014, Xiao and co-workers reported a palladium-catalysed decarboxylation/cycloaddition sequence with vinyl benzoxazinanones as 1,4-dipole precursors [17]. Using this methodology, the authors obtained several *trans* 2-acyl-3-vinyl indolines with excellent diastereo- and enantioselectivity (Scheme 4). The aromatic moieties of the two reaction partners can be modulated, and several ylide-stabilising groups can be used without significant loss of selectivity. The newly formed optically active compounds constitute valuable building blocks and offer straightforward access to complex chiral scaffolds.

**Fig. 2:**
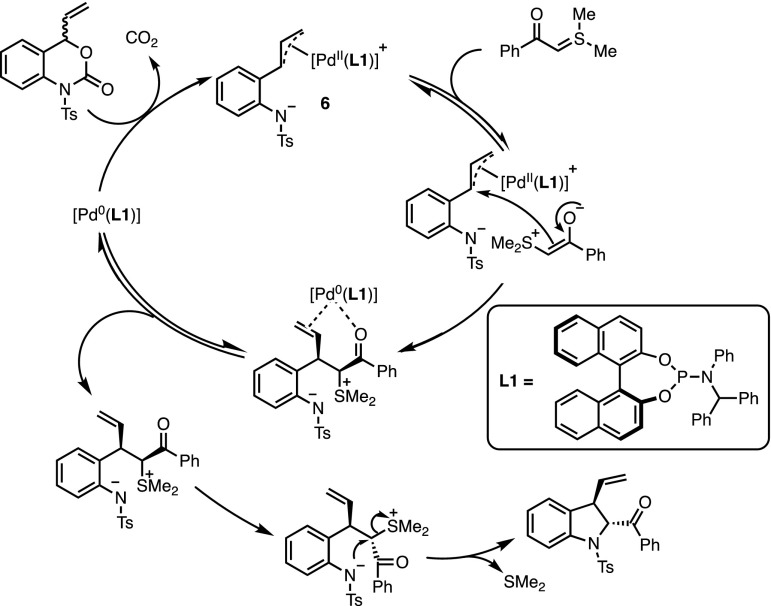
The mechanism of Pd-catalysed decarboxylative formal (4 + 1) cycloaddition

From a mechanistic point of view, the reaction can be compared to a typical Tsuji–Trost-type asymmetric allylic alkylation (Fig. 2). Oxidative addition of the Pd(0) complex into the allylic C–O bond is directly followed by extrusion of CO_2_. The subsequent trapping of the zwitterionic π-allyl intermediate **6** by the ylide constitutes the enantio-determining step of the reaction. Electrostatic interactions between the negatively charged tosyl amide and the sulfonium moiety may explain the exclusive ‘branched’ regioselectivity of the asymmetric allylation. Phosphoramidite **L1** (Fig. 2) proved to be the best ligand to discriminate between the faces of the π-allyl complex. However, the diastereoselectivity is substrate-controlled since indoline derivatives were obtained in a constant > 95:5 diastereomeric ratio regardless of the nature of the chiral ligands. The authors contend that these results suggest that the final nucleophilic substitution of dimethylsulfide requires an initial decomplexation in order to proceed through the least sterically demanding conformation.

**Scheme 5:**
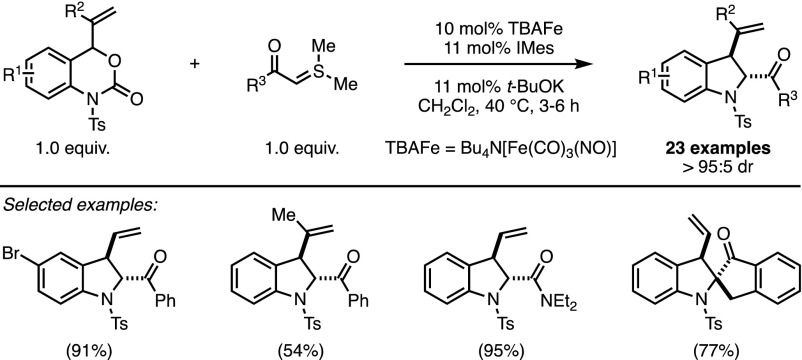
Iron-catalysed decarboxylative formal (4 + 1) cycloadditions. *TBAFe *Bu_4_N[Fe(CO)_3_(NO)]

In 2016, the same reaction was revisited in the context of Fe-catalysis. The interest in this abundant and inexpensive metal has experienced something of a renaissance in recent decades [18]. Notably, nucleophilic iron complexes have emerged as efficient catalysts in several chemical transformations while providing a reduced ecological footprint. A striking example came from the group of Xiao who reported the first iron-catalysed cycloaddition of sulfonium ylides (Scheme 5) [19]. The reaction, albeit not performed in an enantioselective manner, afforded anti-indolines with good yields and excellent diastereocontrol.

**Scheme 6:**
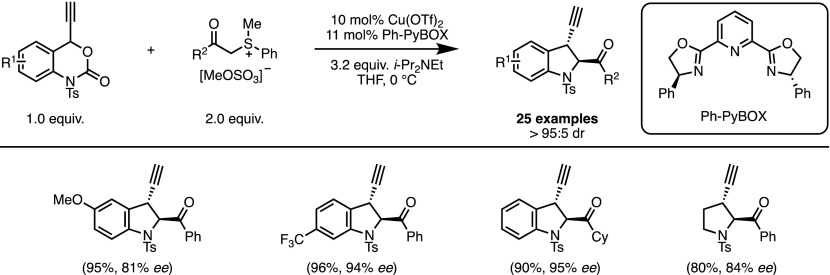
Copper-catalysed decarboxylative formal (4 + 1) cycloadditions. *THF *Tetrahydrofuran

Thereafter, the same group explored the ability of copper-allenylidene intermediates to act as reactive 1,4-dipoles. A novel reaction was then designed in which the vinyl benzoxazinanone functionality was replaced as a propargyl benzoxazinanone (Scheme 6) [20]. Initial studies identified chiral PyBOX ligands as the most efficient in delivering propargyl indolines in good yields with moderate enantiomeric excess. However, the selectivity was dramatically improved when the ylide was generated in situ by deprotonation of the corresponding sulfonium salt with an excess of *N*,*N*-diisopropylethylamine (DIPEA).

**Fig. 3:**
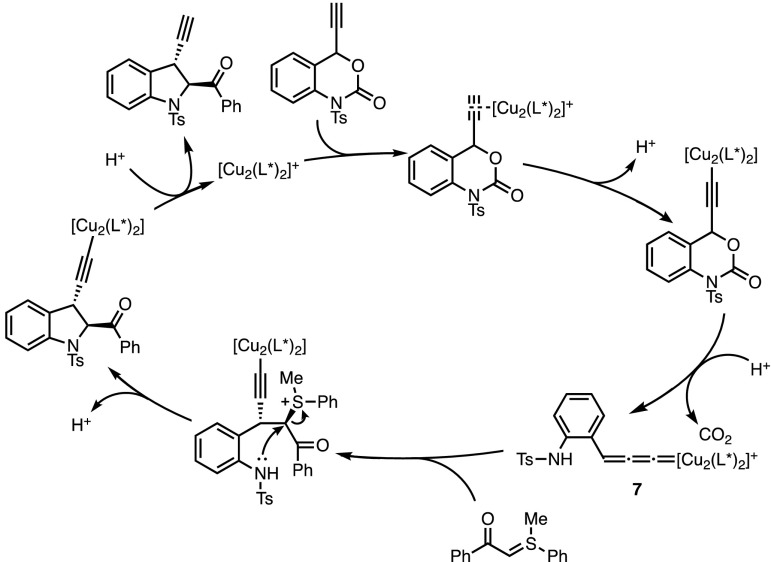
Copper-allenylidene intermediates in decarboxylative formal (4 + 1) cycloadditions

Although the overall transformation appears to be very similar to the palladium-catalysed process reported above, the two reactions involve rather different mechanisms. Notably, the decarboxylation step converts the copper acetylide intermediate into copper allenylidene **7** (Fig. 3). This reactive species then acts as an electrophile [21] in the cycloaddition process. When compared with π-allyl complexes, the origin of the facial discrimination is difficult to establish when considering chiral metal-allenylidenes. However, observed non-linear effects strongly suggest the participation of a multinuclear complex during the enantio-determining step of the reaction. Moreover, mechanistic investigations carried out by van Maarseveen and co-workers on similar allenylidene-based reactions indicate that the presence of DIPEA could help the formation of such multinuclear species [22]. This could explain the remarkable enhancement of enantioselectivity observed when the reaction was performed with an excess of base.

**Scheme 7:**
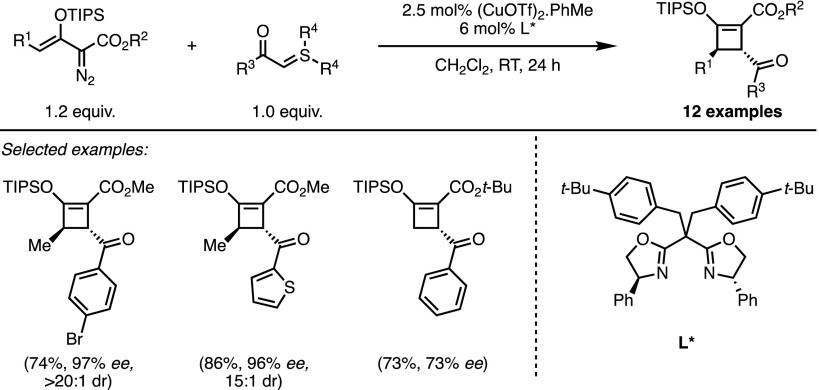
Doyle’s Cu-catalysed asymmetric formal (3 + 1) cycloaddition.* RT* Room temperature

Very recently, an elegant enantioselective copper-catalysed synthesis of optically active cyclobutenes was reported by the group of Doyle (Scheme 7) [23]. The reported strategy relied on a formal (3 + 1) cycloaddition between sulfonium ylides and enoldiazo compounds as 1,3-dipole precursors [24]. A range of cyclobutenes containing two stereogenic centres was accessed in a diastereoselective manner through this method, with a bulky bisoxazoline ligand ensuring good to excellent levels of enantioselectivity.

**Fig. 4:**
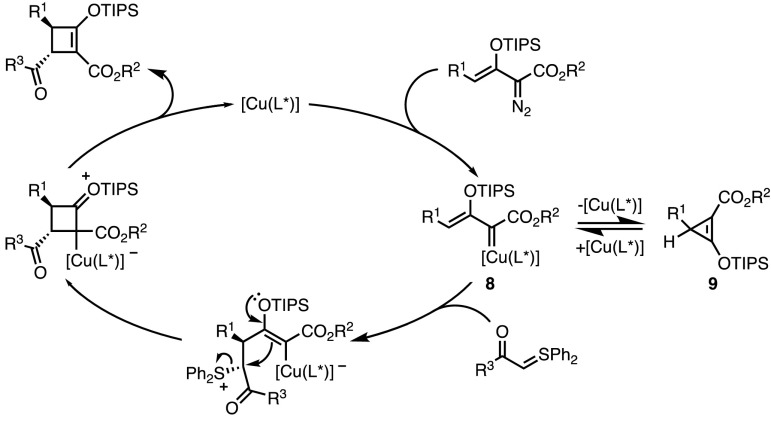
Mechanism of the formal (3 + 1) cycloaddition involving sulfonium ylides

The reaction mechanism proposed by the authors is depicted in Fig. 4. Metal carbenoid **8** was formed by decomposition of the enoldiazo substrate in the presence of the copper catalyst. Based on previous reports, **8** is assumed to be in equilibrium with donor–acceptor cyclopropene **9**. To confirm this assumption, **9** was prepared and submitted to the cycloaddition conditions, whereupon the expected cyclobutenes were isolated with similar yields and selectivity. Nucleophilic attack on **8** by the sulfonium ylide was followed by the cyclisation step. Final decomplexation released the catalyst and the desired cyclobutene.

### Transition Metals as π-Acid Catalysts

**Scheme 8:**
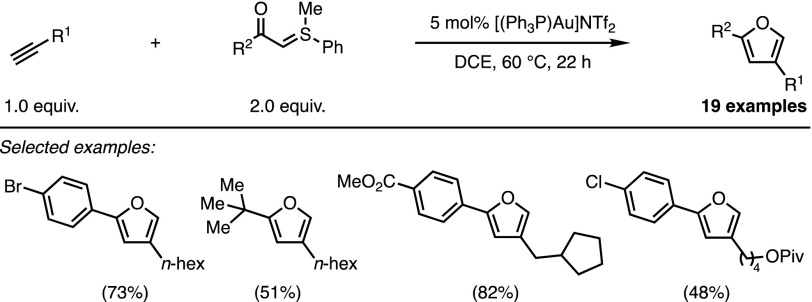
Skrydstrup’s intermolecular furan synthesis.* DCE* 1,2-Dichloroethane

The Corey–Chaykovsky cyclopropanation is perhaps the best-known manifestation of the ability of sulfonium and sulfoxonium ylides to add to Michael acceptors. The pioneering work of Holy and Bolm mentioned above extended this reactivity to various reactive dipoles. Meanwhile, the tremendous development of π-acid catalysis led several groups to envision unreactive π-systems as valuable partners for transition metal-catalysed cycloadditions of sulfonium ylides. In 2012, the groups of Skrydstrup and Maulide independently reported syntheses of furans based on this strategy. In both cases, a cationic π-acidic gold complex promoted a formal (3 + 2) cycloaddition between a stabilised sulfonium ylide and an unactivated alkyne. Unlike the transformations reported in the previous section of this review, sulfonium ylides were now used as three-atom synthons. The intermolecular approach developed by Skrydstrup and co-workers led to the formation of various 2,4-disubstituted furans with good yields (Scheme 8) [25].

**Scheme 9:**
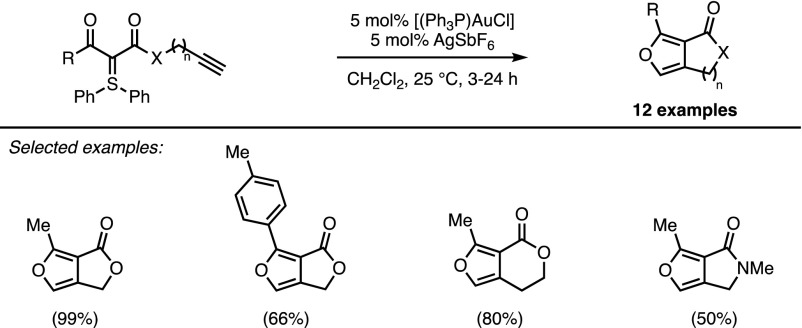
Maulide’s intramolecular furan synthesis

Investigations by Maulide and coworkers focussed on the gold-catalysed conversion of doubly-stabilised sulfonium ylides, prepared via a direct ylide transfer [26], into lactone-fused furan rings [27]. The efficiency of this intramolecular reaction was demonstrated on a wide range of substrates (Scheme 9).

**Fig. 5:**
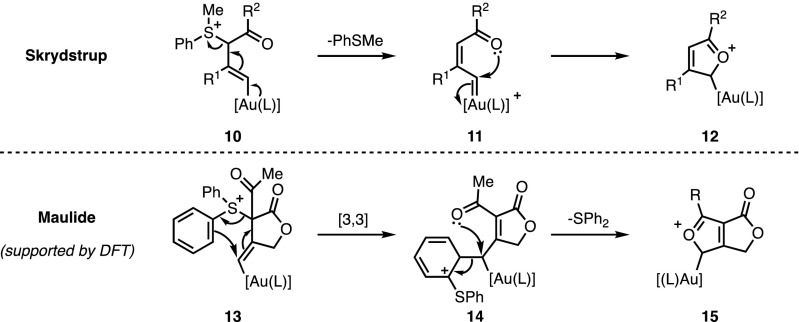
Proposed mechanisms for furan formation

Aside from inter- and intramolecular considerations, these transformations were based on similar systems, which might be expected to react in an identical fashion. Indeed, the formation of a vinyl–gold complex (**10** and **13** respectively), through the addition of the nucleophile onto the activated alkyne, was suggested in the reports from both groups as a reasonable first step (Fig. 5). However, it was at this point that the proposed mechanisms diverged, with the key cyclisation step differing considerably. Skrydstrup and co-workers postulated the participation of the gold-carbenoid intermediate **11**, generated by extrusion of phenylmethylsulfide. The resulting electron-poor metal carbenoid was thought to then undergo an intramolecular attack of oxygen to afford cyclic oxocarbenium **12**. A final aromatising deauration step would release the desired furan moiety. On the other hand, computational experiments undertaken by Maulide and co-workers support a carbenoid-free mechanism, in which vinyl gold complex **13** undergoes a [3,3]-sigmatropic rearrangement leading to intermediate **14**. In this case, the cyclisation step proceeds concomitantly with the extrusion of diphenylsulfide, delivering oxocarbenium **15**, analogous to **12**. Neither route has as yet been conclusively disproved; nevertheless, the computational models could not find an energy minimum corresponding to the free carbene analogous to intermediate **11**.

**Scheme 10:**
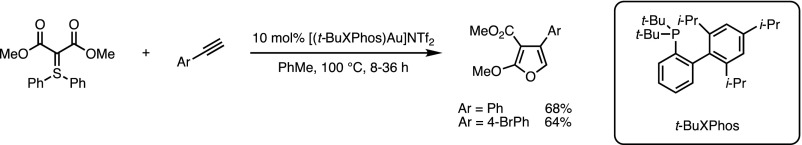
Intermolecular synthesis of trisubstituted furans

**Scheme 11:**
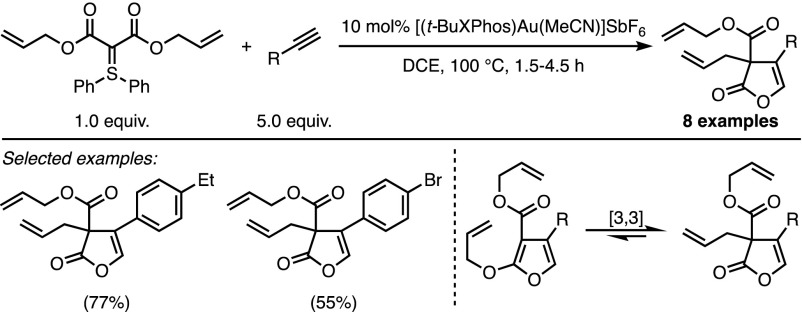
Maulide’s intermolecular furanone formation

In the same study, Maulide and co-workers investigated the behaviour of doubly stabilised ylides in an intermolecular context, analogous to the Skrydstrup procedure. While such reactions resulted in the efficient formation of 2,3,4-trisubstituted furans, higher temperatures were required on account of the lower reactivity of the starting materials (Scheme 10). When allyl esters were used as stabilising substituents, the final product of the intermolecular cyclisation can rearrange through a [3,3] sigmatropic shift, affording dearomatised furanones bearing a quaternary stereocentre (Scheme 11).

During the development of the furanone formation, a side product was detected in which the alkyne coupling partner was not incorporated. By removing the alkyne from the reaction system the allyl-ester derived sulfonium ylides were directly converted into cyclopropane derivatives. This intramolecular gold-catalysed cyclopropanation proved to be remarkably efficient, resulting in good yields and excellent diastereoselectivity and tolerating a broad range of functional groups.

**Scheme 12:**
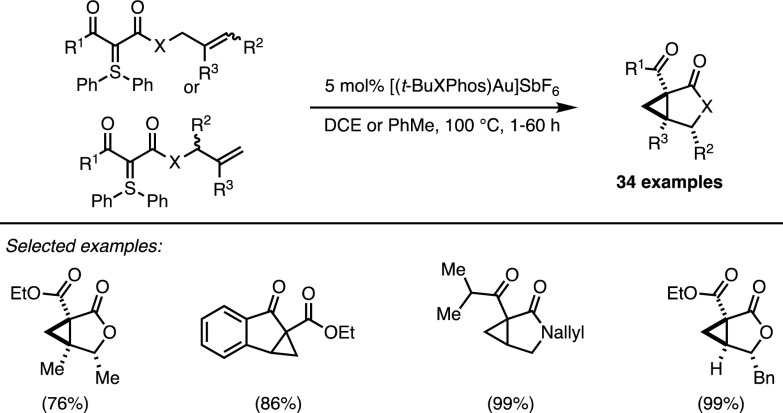
Maulide’s Au-promoted intramolecular cyclopropanation

An intriguing feature of the process was observed during investigations on substituted systems. As depicted in Scheme 12, the position of substituent *R*^2^ in the cyclopropane products is the same, whether starting from the ‘linear’ or ‘branched’ starting material when X = O. This unexpected result suggests a complex underlying mechanism and motivated further mechanistic investigations [28], which ultimately led to the development of an enantioselective version of this reaction [29].

**Scheme 13:**
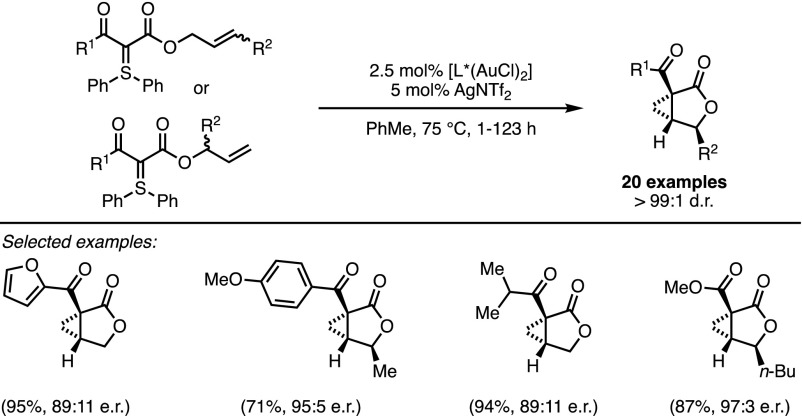
Domino deracemisation/cyclopropanation of sulfonium ylides

The combination of a cationic gold complex and a dimeric TADDOL-based phosphoramidite ligand promoted asymmetric cyclopropanation with high degrees of enantiocontrol and perfect diastereoselectivity. Strikingly, ‘branched’ and ‘linear’ isomers of the starting materials led to the same desired lactone-fused cyclopropanes with high yields and optical purities (Scheme 13).

**Fig. 6:**
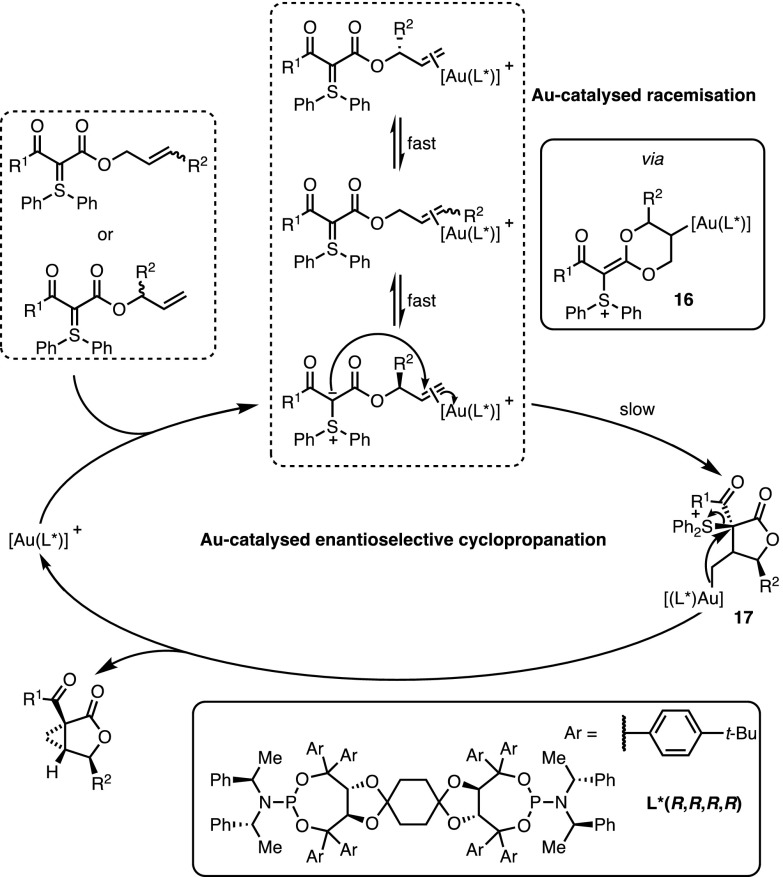
Mechanistic rationale behind the deracemisation/cyclopropanation cascade

The occurrence of a highly unusual (in gold catalysis) deracemisation–cyclopropanation sequence was invoked to rationalise this stereoconvergent outcome (Fig. 6). In the presence of the π-acidic gold catalyst, an equilibrium exists between the ‘linear’ isomer and both enantiomers of the ‘branched’ one. The interconversion of these species occurs through the intermediacy of **16**, which has been directly observed by ^1^H-NMR analysis. Moreover, the chiral catalyst favours the cyclisation of one enantiomer of the ‘branched’ isomer and ensures facial discrimination. Therefore, the whole process constitutes a remarkable example of dynamic kinetic resolution in which the racemisation is dependent on the catalyst. Completing the catalytic cycle, the formation of intermediate **17** was followed by a diastereoselective cyclopropanation with the displacement of diphenylsulfide.

**Scheme 14:**
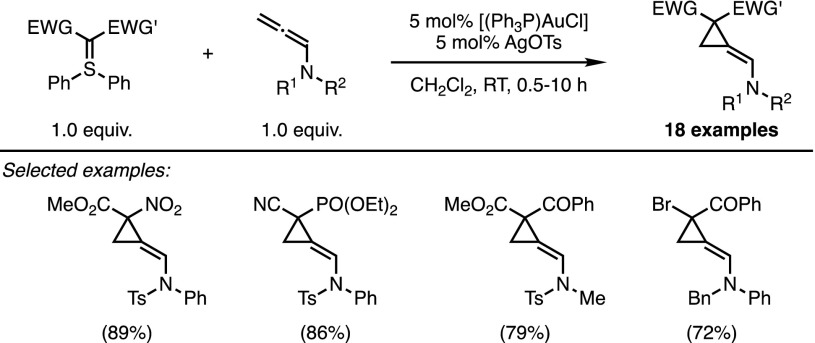
Intermolecular cyclopropanation of allenamides

Interestingly, this novel approach for accessing three-membered rings pointed to new opportunities for diazo-free cyclopropanations. The process, however, proved resistant to various attempts to translate this success into an intermolecular variant, and ultimately the use of electron-rich allenamide derivatives were required for such a process to become feasible, as reported by Maulide and co-workers in 2014 [30]. In the presence of doubly stabilised ylides and a cationic gold complex, *N*-tosylallenamides underwent smooth cyclisations at room temperature (Scheme 14). Computational modelling provided some mechanistic insights to help explain both the regio- and stereoselectivity of the reaction. The most electron-rich double bond co-ordinates the π-acidic catalyst, but the nucleophilic attack occurs on the less sterically demanding terminal position. Additionally, 1,3 allylic strain is responsible for the observed diastereoselectivity and the exclusive formation of (*E*)-olefins. All attempts to perform this reaction enantioselectivity, however, have so far remained unsuccessful.

**Scheme 15:**
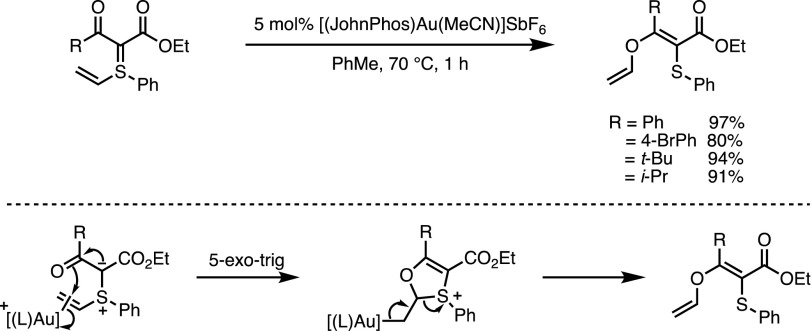
Gold-catalysed [1,4] vinyl migration

In most cases, variations of the substituents on the sulfonium group are of little interest since the corresponding sulfide is necessarily eliminated during the cyclisation step. It has been argued that aromatic substituents play a pivotal role in the mechanism of the furan synthesis reported by Huang et al. (see above text) [27]. In 2016 the Maulide group disclosed the results of their investigations into vinyl-substituted sulfonium ylides. Although Aggarwal had previously built on the ability of vinylsulfonium salts to undergo nucleophilic additions generating versatile sulfonium ylides in situ [31–34], no investigations into vinyl-substituted sulfonium ylides had been reported. When treated with an appropriate π-acidic catalyst, these compounds were shown to efficiently undergo *S*-to-*O* vinyl transfers (Scheme 15) [35]. The reaction resembles a Smiles rearrangement and was proposed to occur via two main steps, namely, an initial attack of the carbonyl oxygen atom onto the activated vinyl substituent in a 5-*exo*-trig manner followed by elimination to generate the product. Alternative mechanistic pathways were ruled out based on the results of isotope labelling experiments. The products were in turn used as substrates in several photocatalytic transformations. Notably, the *S*-to-*O* vinyl transfer was also shown to proceed in the absence of Au, under thermal activation, albeit with much reduced levels of control over the double bond geometry.

### Sulfoxonium Ylides in C–H Functionalisation Processes

**Scheme 16:**
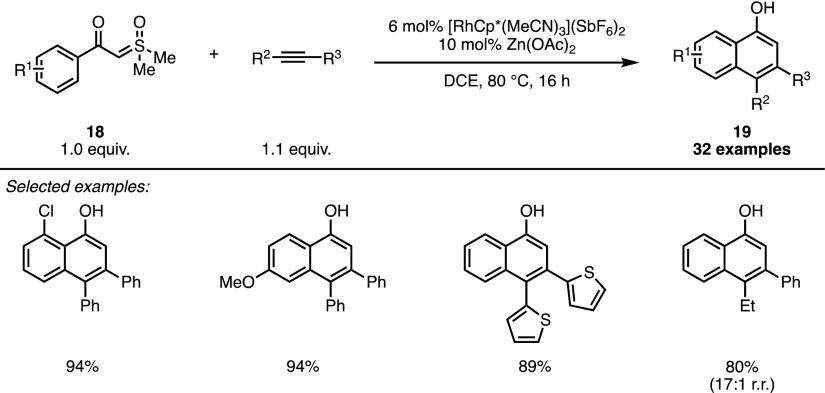
Sulfoxonium ylides as precursors for the Rh-catalysed formation of napthols

C–H functionalisation is an area that, over the last 20 years, has seen unprecedented interest and developments [36]. Techniques based on cyclometallated intermediates have been developed to encompass a broad range of transformations, utilising numerous coupling partners and chelating directing groups. The combination of this ever-growing field with the use of sulfonium ylides as one carbon synthons is one that was, until during the preparation of this review article, completely unexplored. The year 2017 saw the publication of three papers on the subject, two by the laboratory of Li, and one by the laboratory of Aïssa. The first of these papers focussed on the use of stabilised sulfoxonium ylides (**18**) able to direct C–H activation [37]. By combining benzoyl sulfoxonium ylides with alkynes under the action of Rh(III) catalysis, Li and co-workers were able to synthesise a range of naphthol derivatives (**19**) in mostly excellent yields (Scheme 16). Only sterically congested or electron-poor systems afforded yields of < 80%.

**Fig. 7:**
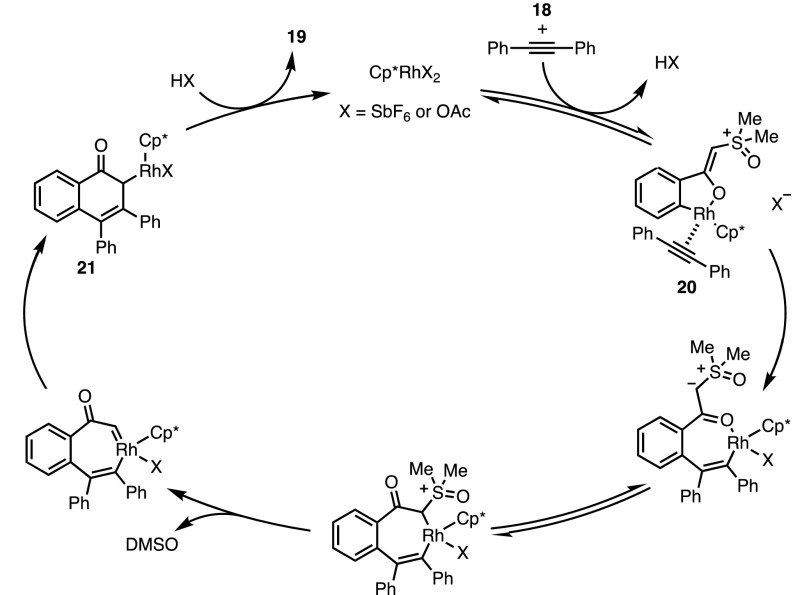
Proposed mechanism of naphthol formation.* DMSO* Dimethyl sulfoxide

The mechanism proposed for this transformation (Fig. 7) involved an initial formation of metallacycle **20**, the formation of which is facilitated by the highly electron-rich, enolate-like delocalised ylide. Following alkyne insertion and the loss of dimethyl sulfoxide (DMSO), migratory insertion affords rhodium enolate **21**, which decoordinates to form the naphthol product **19**. This catalytic cycle is noteworthy as at no point does the metal catalyst undergo formal oxidation or reduction. Most classical, directed C–H functionalisation reactions require either an external or internal oxidant following a reductive elimination step; however, the migratory insertion forming **21** negates the requirement for such an additive.

**Scheme 17:**
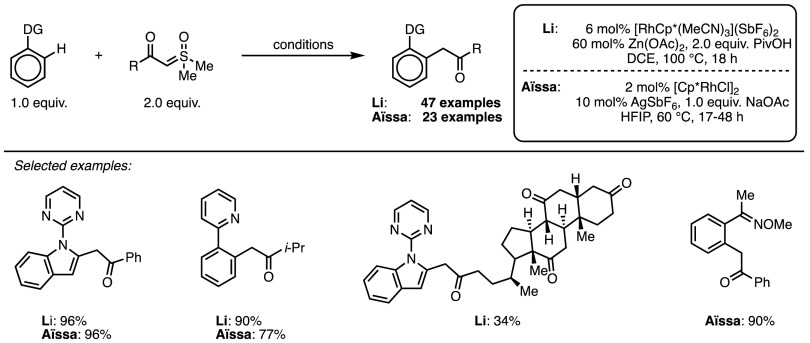
Simultaneously disclosed Rh-catalysed formal C–H insertion reactions.* HFIP* Hexafluoroisopropanol

The other two reports on the use of sulfoxonium ylides for C–H functionalisation processes were published independently and almost simultaneously by the groups of Li [38] and Aïssa [39], and they describe practically identical processes, namely a formal C–H insertion of sulfoxonium ylides into the C–H bond ortho to a directing group (Scheme 17). Such processes have been extensively described in cases where a diazo compound is used as the one-carbon synthon [40], but these publications represent the first use of a sulfur-based ylide for this purpose. The two aforementioned papers describe between them a wide range of combinations of various sulfoxonium ylides with arene and heteroarene partners. While only *N*-based directing groups were reported, the oxime directing group can be hydrolysed for access to *N*-free compounds. Both papers showed derivatisations of the products, of which an Ir-catalysed dehydrative cyclisation to form benz(c)acridines is particularly interesting, making use of the quinoline directing group.

**Fig. 8:**
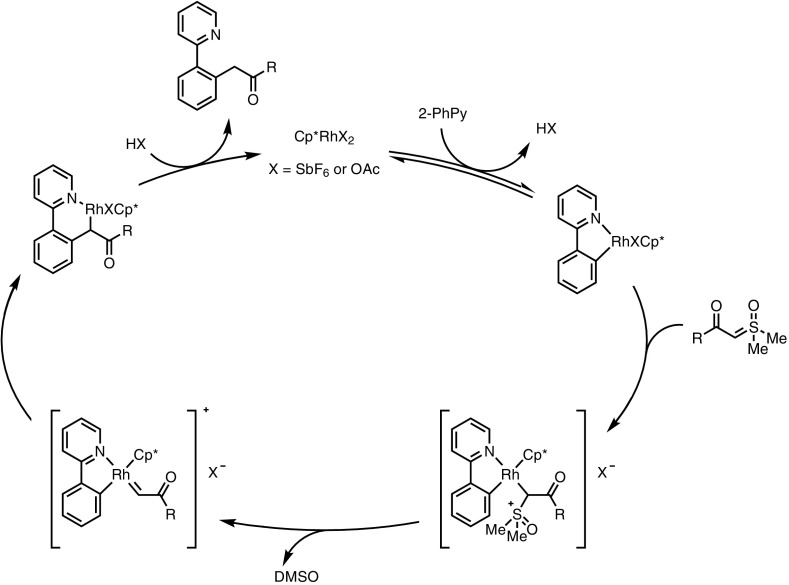
Mechanism of formal C–H insertion of sulfoxonium ylides

As we will see in Sect. 4, sulfur-based ylides have found a wide range of uses as analogues of diazo compounds for the generation of metal carbenoids; however, this is not the mechanism proposed by the authors in this case. In a similar fashion to the previous proposal, a combination of C–H activation (to form a metallacycle) with ylide co-ordination, loss of DMSO and migratory insertion occur without change in the catalyst oxidation state, and thus no external oxidant is required (Fig. 8).

### Sulfonium Ylides as C–S Activation Precursors

**Scheme 18:**
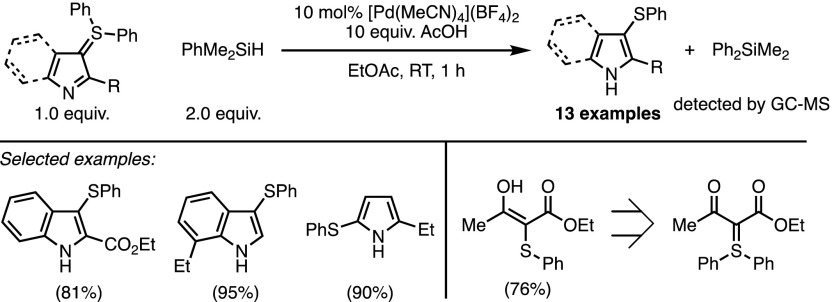
Pd-catalysed sulfur-to-silicon group transfer. GC-MS Gas chromatography–mass spectrometry

In the preceding sections, we have seen how for many years sulfonium ylides have been employed as valuable carbon nucleophiles to achieve C–C bond formation. This reactivity was extensively explored in metal-catalysed transformations in which stabilised ylides displayed a surprising compatibility with Lewis acidic transition metal complexes (Sect. 2/Sect. 3.2). However, the bifunctional structure of sulfonium ylides has been long neglected, and very few metal-catalysed processes directly take place on the sulfonium moiety. In 2013 Maulide and co-workers reported a Pd(II)-catalysed cleavage of non-ylidic Ph–S bonds in various 3-(diphenylsulfanyl)indoles, 2-(diphenylsulfanyl)pyrroles and 1,3-dicarbonyl-derived ylides [41]. The reductive cleavage readily proceeds in the presence of phenyldimethylsilane and affords the corresponding phenylsulfides in good yields (Scheme 18). Unfortunately, the mechanism of this original transformation remains unclear, even though a number of suggestive observations have been made. For example, the transfer of the phenyl group from the sulfur to the silicon atom was confirmed by the detection of diphenyldimethylsilane via GCMS. Deuterium labelling experiments have proven that the reductive process did not incorporate a hydrogen atom from the silane reagent into the final product but that most probably the acetic acid additive acts as the H-atom source instead. Finally, X-ray crystallographic analysis has shown that co-ordination of the N-lone pair to the Pd(II) complex strongly modifies the nature of both the ylidic and neighbouring bonds.

**Scheme 19:**
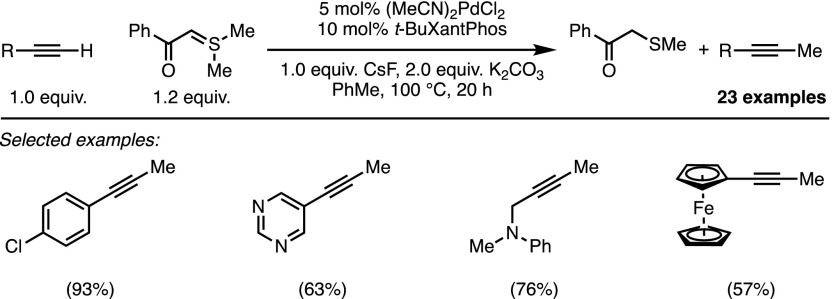
C–S bond activation for the methylation of terminal alkynes

Shortly thereafter, the electrophilic reactivity of dimethylsulfonium ylides was investigated by Li and co-workers who established an elegant palladium-catalysed methylation of terminal alkynes (Scheme 19) [42]. The process can be conceptualised as a Csp–Csp^3^ Sonogashira-type coupling and is effective for a wide range of highly functionalised alkynes. Labelling experiments showed that the methyl group is transferred intact without any exchange of hydrogen atoms, which has led the authors to propose a mechanism proceeding via an initial oxidative addition into the S–Me bond. Although no further evidence is provided to support this proposal, if correct it would represent a remarkable activation mode. Regardless of the pathway involved, the transformation remains interesting in the context of C–S bond activation [43–45].

### Photocatalysis Involving Sulfonium Ylides

**Scheme 20:**
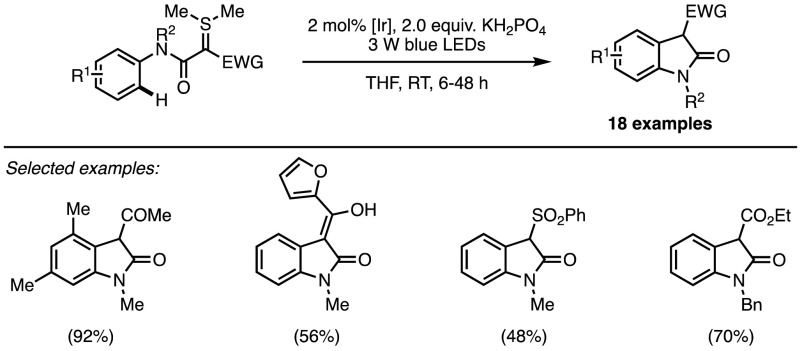
Xiao’s photocatalytic insertion of sulfonium ylides into C–H bonds for oxindole synthesis [48].* LEDs* Light-emitting diodes

**Fig. 9:**
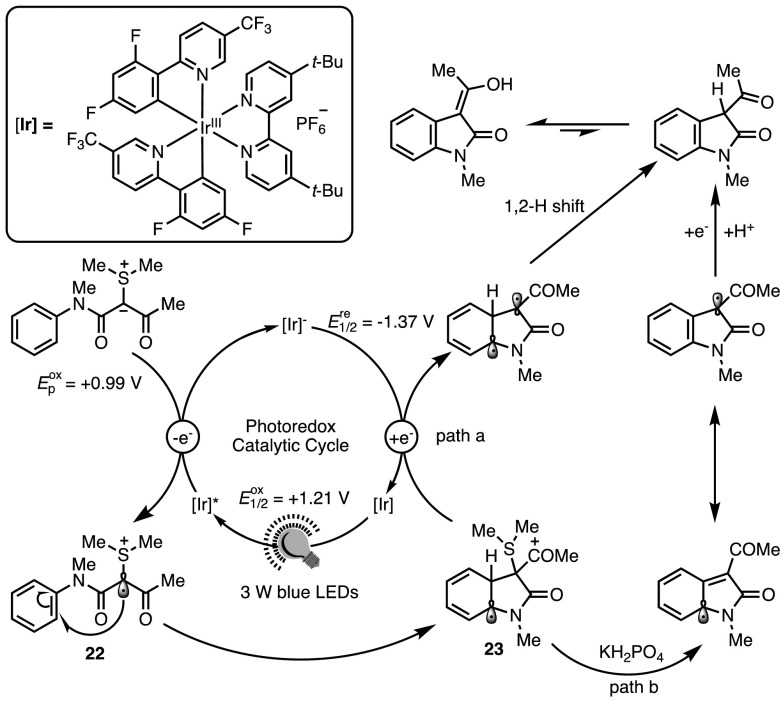
Mechanism of the photocatalysed C–H insertion.* LEDs* Light-emitting diodes

As an alternative to the ionic reactivity paradigm, radical chemistry can be an inexhaustible source of original transformations in organic synthesis. Radicals have an unpaired valence electron and are typically extremely reactive species able to engage in reactions often inaccessible to ionic chemistry. In recent years, radical chemistry has undergone resurgence due to the advent of photocatalysis. This branch of catalysis relies on the ability of appropriate dyes to trigger single electron transfer (SET) processes upon visible light irradiation [46, 47]. A plethora of photocatalytic transformations have been reported in recent years that all involve substrate activation either through single-electron oxidation or reduction by an electronically excited catalyst. With this in mind, it should not be surprising to see the carbanionic character of sulfonium ylides exploited as a valuable tool for developing novel photocatalytic oxidative processes. Nevertheless, only one such report has appeared to date. In 2016, Xiao et al. explored the photocatalytic synthesis of oxindoles in which a doubly-stabilised sulfonium ylide underwent a SET process, thus initiating a formal C–H insertion (Scheme 20) [48]. The reaction occurred in the presence of a cationic complex of iridium(III) that can be excited under blue light irradiation. This excited complex (Ir* in Fig. 9) is a strong oxidant and presumably able to abstract an electron from the sulfonium ylide to form radical cation **22**. It is worth mentioning at this point that it might be expected that energy transfer from the excited photocatalyst could result in decomposition of the ylide to form the free carbene [49]; no such process is, however, observed under the reported conditions. Formation of five-membered intermediate **23** occurs rapidly, followed by termination through either pathway a or b.

**Scheme 21:**
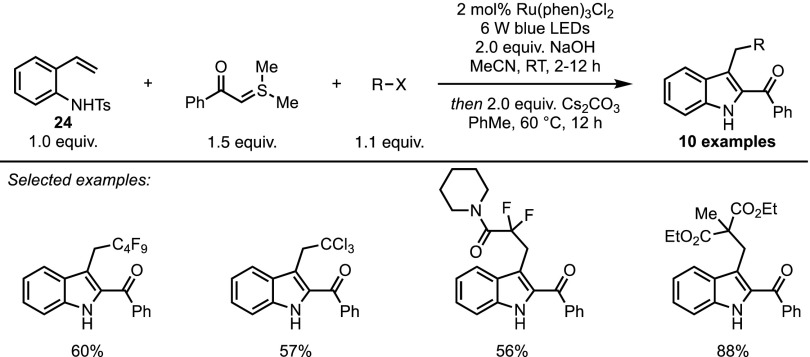
Photocatalytic synthesis of 2,3-disubstituted indoles

Shortly before the publication of this review, the group of Xiao reported a photo-induced synthesis of 2,3-disubstituted indoles from sulfonium ylides, *N*-tosyl vinylaniline **24** and a radical precursor. This transformation relies on a blue light-driven formal (4 + 1) cycloaddition and can be promoted through the use of a number of radical sources. Indeed, trifluoroethylated indoles were readily obtained when using Umemoto’s reagent in a remarkable catalyst-free procedure. Other radical precursors, however, required the use of Ru(phen)_3_Cl_2_ as a photocatalyst to generate the cyclised products, which could be obtained in moderate to excellent yields (Scheme 21).

**Fig. 10:**
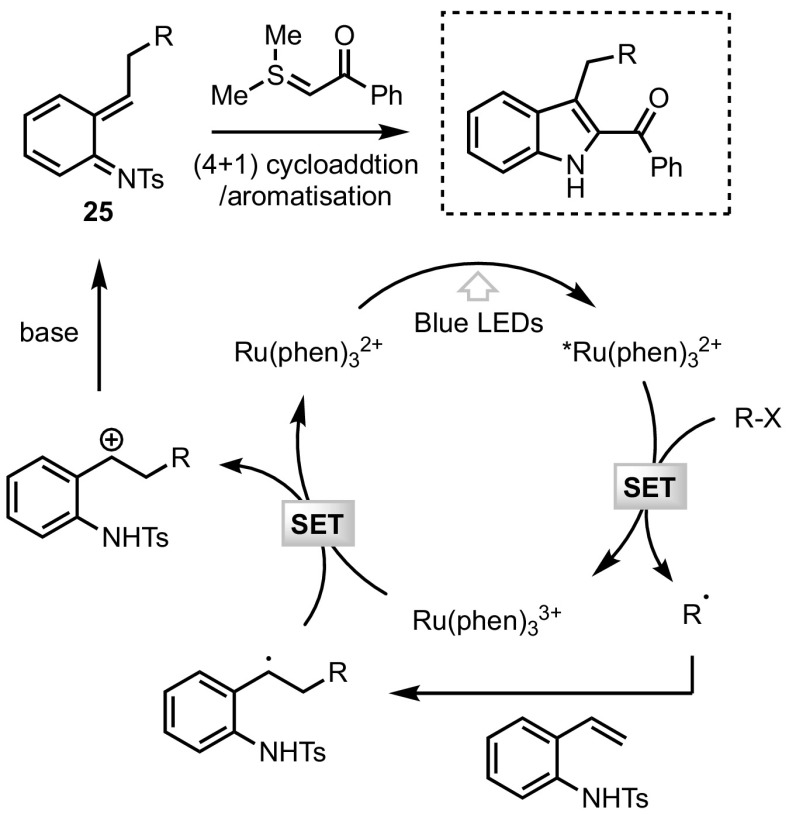
Mechanism of the photocatalysed synthesis of 2,3-disubstituted indoles

In contrast to the previous example described in this section, the SET process does not directly involve the sulfonium ylide reagent. The authors suggest that the initial radical species adds onto the styrene functionality of **24**, and the resulting radical is then oxidised through a second SET to provide an aza ortho-quinone methide **25** (Fig. 10). This 1,4-dipolar intermediate is then trapped by the sulfonium ylide in a classical cycloaddition process.

## Sulfonium and Sulfoxonium Ylides as Metal-Carbene Precursors

We have seen in the previous section how sulfur-based ylides have been used as carbenoid/one-carbon synthons in a number of transition metal-mediated processes. One of the most ubiquitous methods of introducing such synthons is through the action of metal carbenoids (also known as alkylidenes), generated from diazo compounds and metal complexes. Typical reactions include cyclopropanation and insertion into C–H and X–H (X = N, O, S, P, Se) bonds [50–54]. While a highly popular and efficient reaction choice, the use of diazo compounds on a large scale does have significant drawbacks for both safety and operational simplicity. A number of sulfonium and sulfoxonium ylides have therefore been investigated as alternative metal-carbenoid precursors [55]. Although such approaches do offer notable improvements, the ease with which the reverse reaction occurs, i.e. the formation of sulfonium ylides from the combination of metal carbenoid species with sulfides (see Sect. 5), is a major hurdle to overcome.

### Cyclopropanation Reactions

**Scheme 22:**
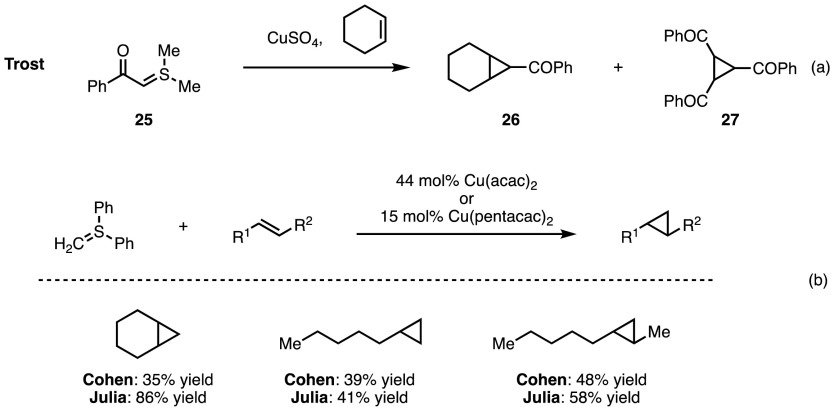
Initial investigations towards the use of sulfonium ylides as diazo surrogates

In 1966, Trost published an example of copper-catalysed cyclopropanation employing sulfonium ylide **25** (Scheme 22a) [49]. However, the expected cyclopropane **26** was only isolated in trace quantities; instead, cyclopropane **27** was the major product, representing the cyclopropanation of a carbene dimerisation product. More successful were the Cu(II)-catalysed attempts of Cohen and co-workers in which the expected cyclopropane could now be isolated in an appreciable yield when using diphenylsulfonium methylide as the carbenoid source [56]. Under these conditions no trimers analogous to **27** were observed. Julia et al. reported an improved procedure in which they exchanged the acac ligand for the more bulky pentacac (Scheme 22b) [57]. This enabled lower catalyst loadings to be employed and also resulted in a significant increase in yield for a handful of cases.

**Scheme 23:**
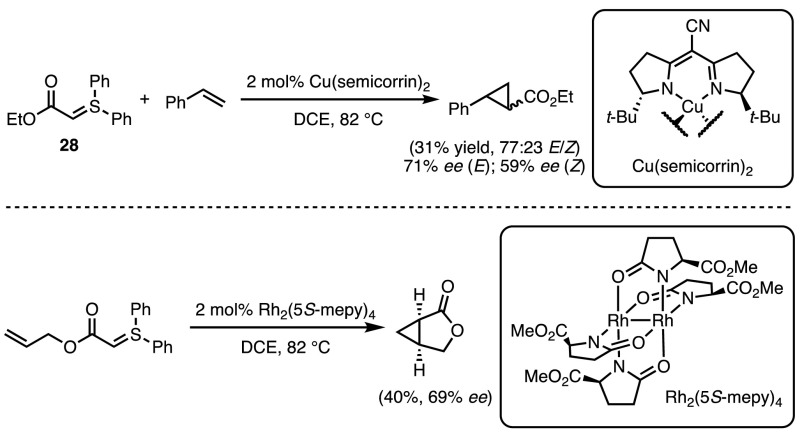
Asymmetric metal-catalysed cyclopropanations

It was not until 1999 that the first enantioselective process was developed by Müller and co-workers [58]. By using either a Cu(I) or Rh(II) chiral catalyst, ylide **28** could be used to generate cyclopropanes in moderate yields (Scheme 23). A direct comparison of diazo compounds with sulfonium ylides was also undertaken, which provides insight into the mechanism at work. While for each case the yields of the diazo-employing reactions were observed to be higher, reflecting their higher reactivity, the diastereo- and enantioselectivities were almost identical. This suggests that the key enantiodetermining steps are likely the same and supports the existence of an intermediate metal-carbenoid.

**Scheme 24:**
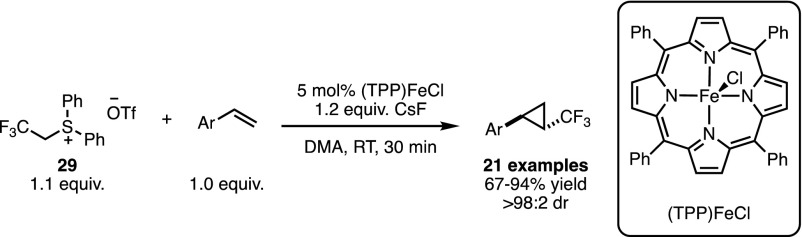
Iron-catalysed carbenoid transfer to generate CF_3_-substituted cyclopropanes.* DMA* Dimethylacetamide

Further breakthroughs have only been achieved in recent years, with the use of iron porphyrin catalysts. In 2016 the Gu group reported the formation of trifluoromethyl cyclopropanes via the cyclopropanation of styrenes using the ylide generated from sulfonium salt **29** (Scheme 24) [59]. Good trans-selectivity was observed across a broad range of styrenes, however no alkyl-substituted, or 1,2-disubstituted alkenes were reactive under these conditions. The (TPP)FeCl catalyst significantly outperformed previously reported systems, with Cu(acac)_2_ able to achieve a 16% yield, and copper sulfate and rhodium diacetate completely inactive.

The synthesis of difluoromethyl cyclopropanes can also be achieved using the same catalyst, as reported in 2017 [60]. A 20 mol% of Zn dust was added to assist the reduction of Fe(III) to Fe(II), believed to be the active cyclopropanation catalyst.

### Insertion Reactions into X–H Bonds

**Scheme 25:**
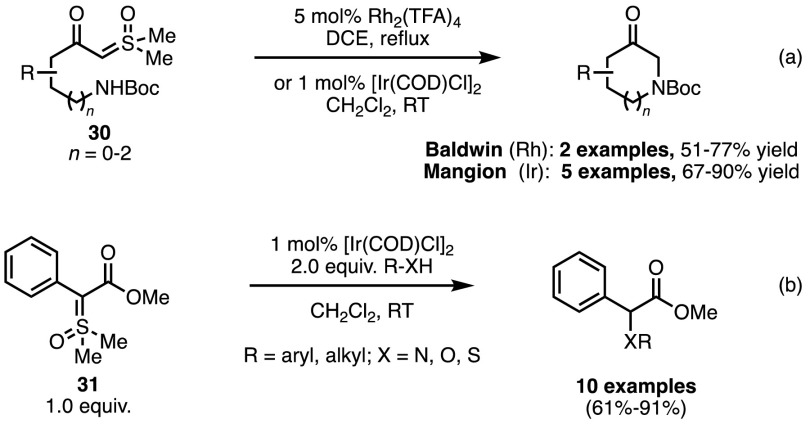
Rhodium- and iridium-catalysed X–H bond insertion reactions

In addition to cyclopropanation, metal carbenoids are also known for their insertion into polar X–H bonds. Interestingly, for such processes utilising sulfur-based ylides as precursors, the literature focusses solely on the use of sulfoxonium ylides, a species we have yet to thoroughly discuss in this review. In classical chemistry these compounds are often employed when extra ylide stability would be beneficial to the reaction. Their first use in transition metal-catalysed X–H bond insertion was reported in 1993 by Baldwin and co-workers (Scheme 25a) [61]. Ring opening of lactams with dimethylsulfoxonium methylide was shown to lead to β-keto sulfoxonium ylides of type **30**. Upon exposure to 5 mol% of Rh_2_(TFA)_4_ the in situ-formed metal carbenoid underwent a rapid N–H insertion to generate a six-membered ring in 77% yield. While this reaction was efficient under these conditions, the Cu catalysts discussed in Scheme 22 were no longer competent for this process. The extension of this method to γ-lactams was also evaluated and 5-oxopipecolic acid was produced in 51% yield.

The efficiency of these reactions drew interest to the extent that Merck & Company began their own research programme, and in 2009 Mangion and co-workers published a more general methodology, based on Baldwin and co-workers’ original conditions [61]. By treating ylides of type **31** with [Ir(COD)Cl]_2_ Merck researchers were able to achieve intermolecular X–H insertion reactions with a wide range of coupling partners (Scheme 25b) [62]. When Rh_2_(TFA)_4_ was used as the catalyst for the reaction of **31** and aniline, only 22% of the desired product was obtained. As a rationale for this observation the authors proposed that the DMSO released after formation of the metal-carbenoid poisoned the catalyst, an assertion that was reinforced by relevant control experiments. With [Ir(COD)Cl]_2_ it was possible to react the sulfoxonium ylides with a range of primary and secondary amines, alcohols and thiols to afford insertion products in yields ranging from 63 to 93%. The substrates previously investigated by Baldwin were also reactive under these conditions, generating various cyclic structures without the requirement for high reaction temperatures or for catalyst loadings of > 1% (Scheme 25a).

Further studies proved that Au and Pt salts were also competent catalysts for these transformations [63]. AuCl(SMe_2_) proved to be the most effective catalyst (94% yield for the reaction of **31** with aniline), with Pt(COD)Cl_2_ and AuCl_3_ also proving satisfactory (yields of 81 and 78% respectively, compared with 91% for [Ir(COD)Cl]_2_). NMR spectroscopy was used to provide evidence for the existence of iridium carbenoids. However, such species were not observed in the gold-based systems, which the authors attribute to the short lifetimes of gold carbenoids, and therefore proposed analogous reaction mechanisms for the two processes.

**Scheme 26:**
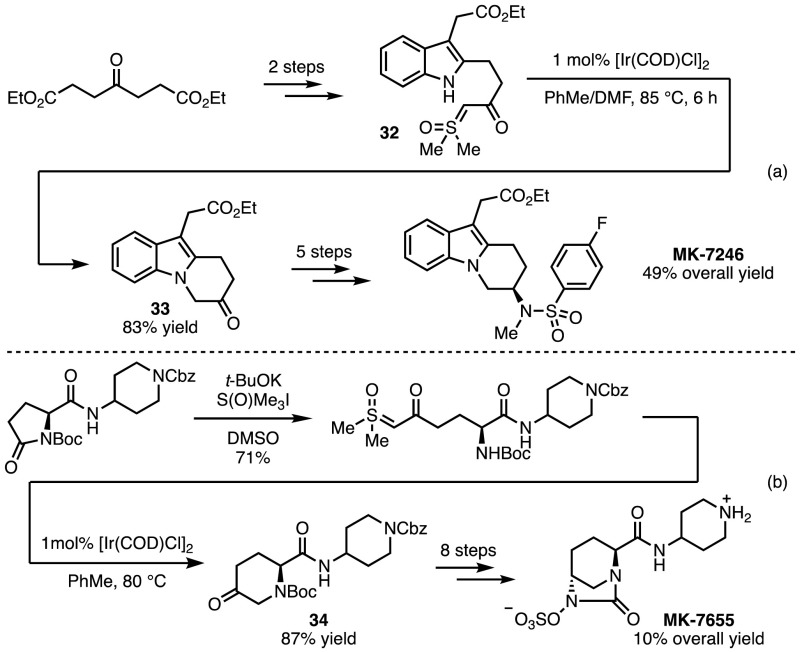
Iridium-catalysed N–H insertion in the synthesis of biologically relevant targets.* DMF* Dimethylformamide

By way of application, a number of Merck lead compounds were synthesised relying on X–H insertion key steps. MK-7246, a selective CRTH2 antagonist with potential for the treatment of respiratory disease, was accessed in a short sequence of eight steps (Scheme 26a) [64]. The key Ir-catalysed intramolecular N–H insertion step converted **32** into **33** in 83% yield. Once optimised, the synthesis was scaled up to multi-ton-scale in a pilot plant, and the sequence required no chromatographic purification.

The synthesis of MK-7655, a β-lactamase inhibitor, was similarly achieved within the Merck process group (Scheme 26b) [65]. For the key N–H insertion step, iridium-based catalysts were again found to be the most suitable, and **34** was produced in 87% yield.

**Scheme 27:**
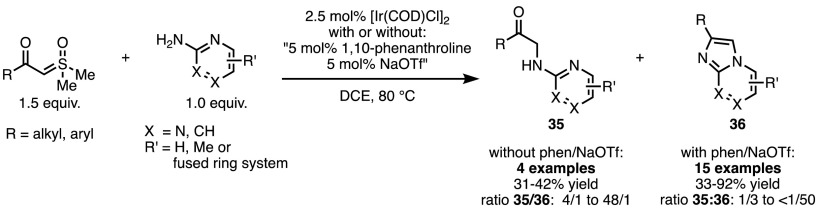
Ligand effects in X–H insertion reactions

Forays have also been made into the realm of heterocycle synthesis by the groups of Shekhar and Hopmann. [Ir(COD)Cl]_2_-catalysed insertion of sulfoxonium ylides into the N–H bond of 2-aminopyridine derivatives afforded the products (**35**) that one might expect from an N–H insertion reaction (Scheme 27) [66]. More interestingly, when 1,10-phenanthroline and NaOTf were added as ligand and additive, respectively, the key iridium carbenoid preferred to react with the pyridine lone pair, rather with than the free NH_2_, to afford azole **36**.

**Scheme 28:**
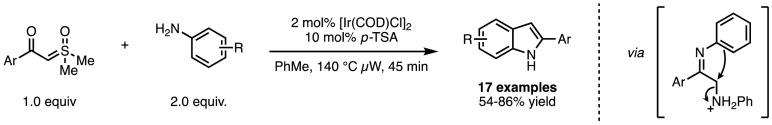
Hopmann’s N–H insertion for indole synthesis.* p-TSA p*-Toluenesulfonic acid

Hopmann’s group later showed that Ir-catalysed N–H insertion of sulfoxonium ylides into anilines could be used for the synthesis of indoles (Scheme 28) [67]. A cascade of carbenoid formation and N–H insertion followed by acid-catalysed imine formation, substitution and aromatisation was active, explaining why the aniline involved in the N–H insertion step was not incorporated into the final product.

### Insertion Reactions into C–H Bonds

**Scheme 29:**
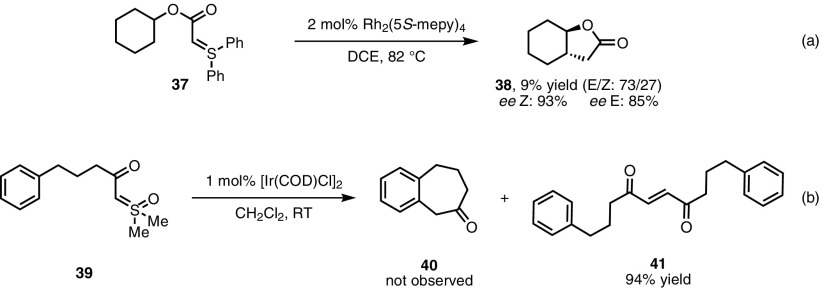
Limitations in C–H insertion reactions

The insertion of diazo-derived metal carbenoids into C–H bonds is a well-understood and synthetically useful transformation [52]. An analogous reaction employing sulfonium/sulfoxonium ylides would thus be a valuable addition to the synthetic toolkit. Unfortunately, only a handful of examples of such reactivity can be found in the synthetic literature. Only a single transformation has been reported using sulfonium ylide precursors, detailing the reaction of ylide **37** under rhodium catalysis (Scheme 29a) [58]. The major product of the reaction was not in fact the desired lactone (**38**), which was isolated in 9% yield, but the product of carbene dimerisation. This outcome was again observed when sulfoxonium ylide **39** was exposed to iridium catalysis by Mangion et al. [62]. When the diazo analogue of **39** was employed, the major product observed was cycloheptanone **40**. In contrast, sulfoxonium ylide **39** led exclusively to the dimerisation product **41** in 94% yield (Scheme 29b). The working hypothesis remains that the reaction of metal carbenoids with remaining ylide starting material is kinetically more favourable than C–H insertion; conversely, C–H insertion is competitive with the reaction of the carbenoid with a diazo compound.

**Scheme 30:**
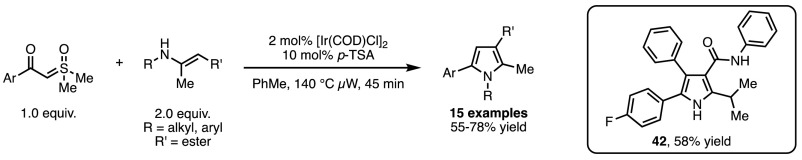
Iridium-catalysed C–H insertion for pyrrole synthesis

The sole successful example of C–H insertion pertinent to this review was reported in 2017 by Hopmann and co-workers [67]. By combining enamines with sulfoxonium ylides, it was possible, through the action of an iridium catalyst, to afford the products of C–H insertion, which in the presence of an acid additive readily cyclised to form highly substituted pyrroles (Scheme 30). The potential of the methodology was showcased through the synthesis of the pyrrole subunit (**42**) of atorvastatin (Lipitor), a cholesterol-lowering drug.

That there only exists one successful case of formal C–H insertion is a clear indication that such processes can be regarded as the poor cousin of the N–H insertion and cyclopropanation reactions in cases when sulfur-based ylides are used as the precursor. The increased nucleophilicity of the ylides relative to diazo compounds is likely to be the key challenge, as nucleophilic attack of the ylide onto the electrophilic carbenoid can be seen to outcompete C–H insertion processes.

### Miscellaneous Metal Carbenoid Reactions

A number of stable metal carbenes have long been used as catalysts for a range of reactions, not least the olefin metathesis reaction. As described so far in this section, sulfonium ylides have been used as metal carbenoid precursors for a number of onward reactions, but it was not until the early 2000s that this approach was used for the synthesis and isolation of such complexes [68, 69]. In studies by Milstein et al., this technique was applied on a number of metal complexes, including those of rhodium, iridium, ruthenium and osmium, as well as on a number of alkylidene units. Initially carried out using sulfonium ylides in solution, more recent investigations have centred on the use of polymer-supported sulfonium ylides, enabling the reuse of the sulfide carrier. Using this method, which relies on the reactivity of diaryl sulfides, the first-generation Grubbs’ catalyst and Werner’s carbene could both be prepared.

Sulfonium ylides have also been used in metal-catalysed polymerisation processes. De Bruin and co-workers found that when exposed to rhodium catalysis, dimethylsulfoxonium methylide polymerised to polymethylene in yields varying between 15 and 80% [70]. The co-polymerisation of sulfonium ylides with diazocompounds was also reported using the same catalyst.

## Cascade Reactions Involving Transition Metal-Catalysed Ylide Formation

Classical routes to sulfur-containing ylides mostly revolve around the deprotonation of the corresponding sulfonium/sulfoxonium salt, usually generated via alkylation. Although conceptually simple, this approach has, by its very nature, major limitations. Prominent among these is the incompatibility of base-sensitive functionalities. The choice of base is also an important consideration, as sulfonium salts have long found employment as alkylating reagents: a non-nucleophilic base is therefore essential. The final problem, albeit not limited to deprotonation-based approaches, is the potential for chemoselectivity issues if there are multiple protons with similar pKa values. In order to simplify matters, most sulfonium/sulfoxonium salt precursors either contain solely degenerate substituents (e.g. trimethylsulfoxonium) or direct the deprotonation through the use of one or more electron withdrawing groups. Such groups also help to stabilise the ylide thus generated, facilitating controllable onward reactions.

**Scheme 31:**
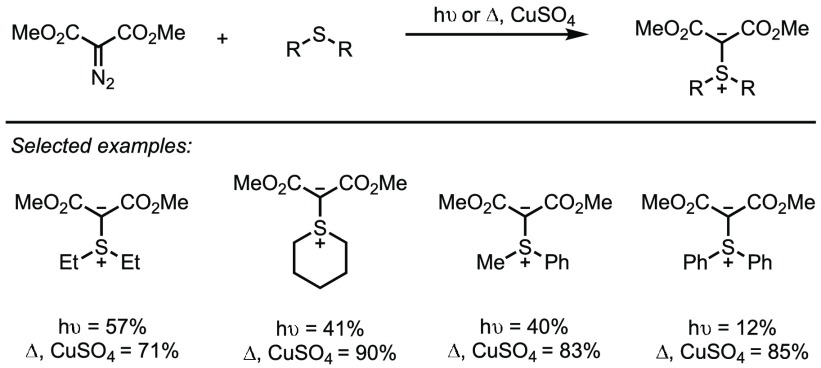
Thermal versus photochemical formation of sulfonium ylides

Efforts to achieve alternative preparations circumventing these issues have been at the centre of sulfur-ylide research for some time. One such strategy is the reaction of a sulfide with an electron-poor carbenoid intermediate to afford the desired ylide [55, 71]. First reported by Diekmann in 1965, the photochemical decomposition of bis(phenylsulfonyl)diazomethane in sulfur-containing solvents, such as dimethylsulfide, dibutylsulfide and dimethylsulfoxide, was shown to afford ylides in moderate to good yields (Scheme 31) [72].

An alternative route, utilising metal carbenoid intermediates emerged shortly thereafter, in which Ando and co-workers employed copper sulfate as a precursor (Scheme 31) [73, 74]. As the metal carbenoids have lower energy than photolytically generated carbenes, the yields for this process were uniformly higher, many proceeding in excellent yields. The avoidance of UV-light, always an issue with scalability and potentially safety, was a further advantage.

The group of Porter showed in 1978 that rhodium acetate could serve as a replacement for copper sulfate in the synthesis of sulfonium ylides [75]. As a result of the high efficiency of rhodium salts in the decomposition of diazo compounds, only 0.12 mol% of catalyst was needed to deliver the desired thiophenium ylide in 95% yield, compared to 35% when using copper catalysis at reflux for 8 days.

**Fig. 11:**
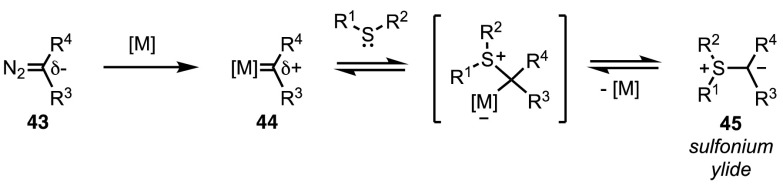
General mechanism for the metal-catalysed synthesis of sulfonium ylides from diazo compounds

The mechanism of this reaction can be envisaged as a double umpolung of the carbenic carbon atom (Fig. 11). Decomposition of nucleophilic diazo compound **43** in the presence of a metal complex leads to electrophilic metal carbenoid **44**. This intermediate can undergo nucleophilic attack from sulfides. The ylide (**45**) thus formed is again nucleophilic at the carbon atom.

As a result of the mild reaction conditions and good chemoselectivity displayed, the transition metal-mediated generation of sulfonium and sulfoxonium ylides has rapidly been adopted by the synthetic chemistry community as one of the methods of choice for the generation of such compounds. The transformations are in fact so clean that a number of onward reactions, using in situ formed sulfonium ylides in domino processes, have been developed. Depending on the structure of the ylide, a number of transformations can occur such as [2,3]-sigmatropic rearrangements, 1,2-migrations and Corey–Chaykovsky-type reactions. These processes have been summarised in a number of review articles [76–78], and the purpose of this section of our review is to provide an overview of significant and recent examples.

### [2,3]-Rearrangement of Sulfonium Ylides

**Fig. 12:**
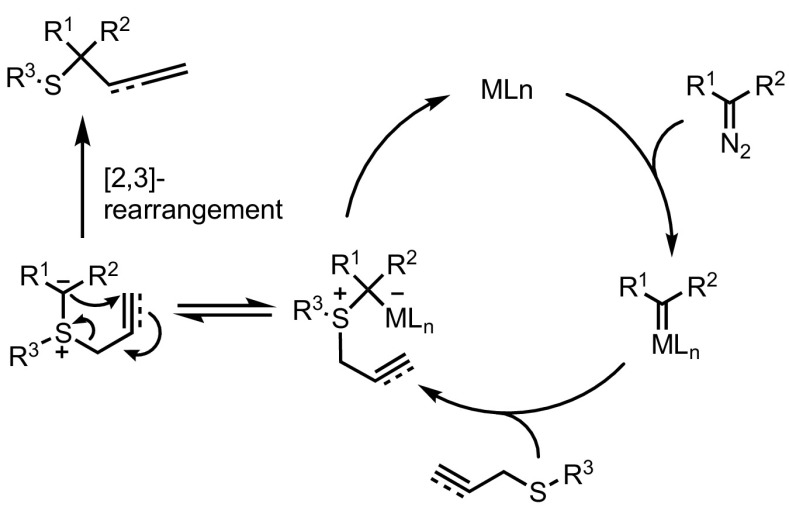
General proposed mechanism for the Doyle–Kirmse reaction

Sigmatropic rearrangements have long been known to be powerful tools for the efficient formation of C–C bonds, and [2,3]-sigmatropic rearrangements are no exception. Significant acceleration has been observed when one or more bonds in the system are highly polarised; the fastest reactions occur when an atom bears a formal charge. In this context, it is not surprising that sulfonium ylides have proven to be useful substrates for these reactions. Given the aforementioned disadvantages in preparation and deprotonation of complex sulfonium salts, the in situ formation of sulfonium ylides through diazo decomposition followed by subsequent rearrangement rapidly emerged as a synthetically appealing approach. The reaction of metal-carbenoid intermediates with allyl or propargyl sulfides, followed by a spontaneous [2,3]-rearrangement of the zwitterionic intermediate is known as the Doyle–Kirmse reaction (Fig. 12) [79, 80]. This transformation has been studied extensively and offers a unique approach for the synthesis of *S*-substituted quaternary centres.

**Scheme 32:**
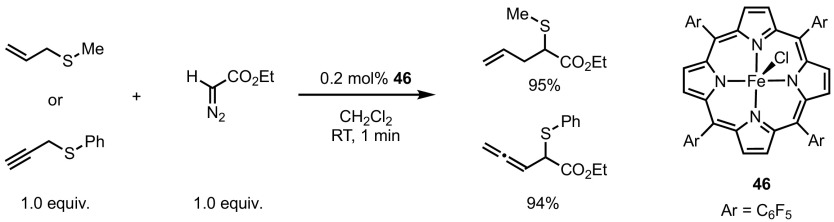
Iron-catalysed Doyle–Kirmse reaction of allyl and propargylsulfides

**Scheme 33:**
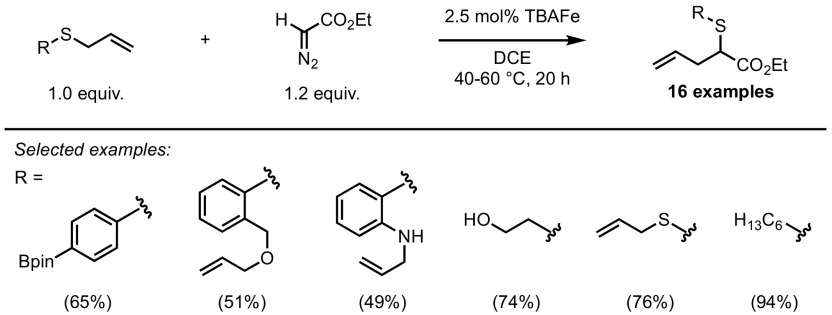
Highly chemoselective TBAFe-catalysed Doyle–Kirmse reaction

As with many classical diazo derivatisations, copper and rhodium salts have been extensively studied due to their ability to catalyse such processes without the need of complex ligand architectures. Nonetheless, a broad range of catalysts, based on different metals, such as Co [81], Ag [82], Pd [83], Ru [84–86] and Fe [87–90], have also been studied in the Doyle–Kirmse reaction. The diversity of catalysts and conditions has led to a broad range of applications on a multitude of substrates. Typically, allylsulfides and propargylsulfides are converted to homoallyl- and homoallenylsulfides, respectively, as demonstrated, for example, by the iron porphyrin-catalysed process depicted in Scheme 32 [91]. Furthermore, the high efficiency of catalyst **46** [similar to (TPP)FeCl, previously encountered in Scheme 24] resulted in catalyst loadings as low as 0.2 mol% under the given reaction conditions. Homoallyl and homoallenyl sulfides could be obtained in excellent yields (from allyl and propargyl sulfides, respectively) at room temperature in very short reaction times. Another compelling aspect of the Doyle–Kirmse reaction is its high functional group tolerance. When using TBAFe as a catalyst, the reaction was performed chemoselectively in the presence of boronates, allyl ethers, secondary anilines, disulfides and free alcohols (Scheme 33) [92]. Interestingly, and unusually for such catalysts, the metal complex used in this reaction is electron-rich and decomposes the diazo compound via a nucleophilic attack.

**Scheme 34:**
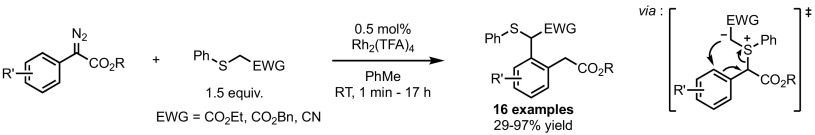
Rhodium-catalysed thia Sommelet–Hauser rearrangement of benzylsulfides

It is also possible to promote [2,3]-sigmatropic rearrangements in which the π-component is embedded in an aromatic system. In such cases, the reaction is better known as the Sommelet–Hauser rearrangement. The first rhodium-catalysed thia-Sommelet–Hauser reaction between diazo(aryl)acetates and arylsulfides was reported by Wang’s group in 2008 (Scheme 34) [93]. The mechanism involves an unusual ylide transposition, as the initially formed, highly stabilised sulfonium ylide undergoes a reversible proton transfer followed by [2,3]-sigmatropic rearrangement. Despite the required dearomatisation step, the reaction proceeds smoothly at room temperature and delivers di- and trisubstituted arenes through selective *ortho*-substitution.

**Scheme 35:**
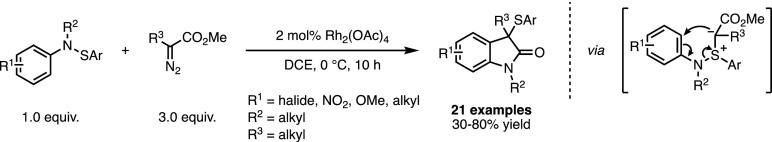
Wang’s modified Gassman oxindole synthesis

In 2011, Wang‘s group applied this methodology to the synthesis of oxindoles, though a modification of Gassman’s synthesis, triggered by Rh-catalysed decomposition of diazo compounds in the presence of sulfenamides (Scheme 35) [94]. Through this method, oxindoles carrying a quaternary centre were synthesised in one step in moderate to good yields.

Generally, the [2,3]-sigmatropic rearrangements of ylides result in the formation of chiral products. The development of an enantioselective variant would be highly appealing, as it would allow the stereocontrolled formation of a C–S and a C–C bond to the same carbon atom. Concerted efforts towards this goal have spanned the last two decades and predominantly focussed on the use of chiral metal complexes. However, only recently have good enantioselectivities in purely catalyst-controlled systems been achieved [95].

**Scheme 36:**
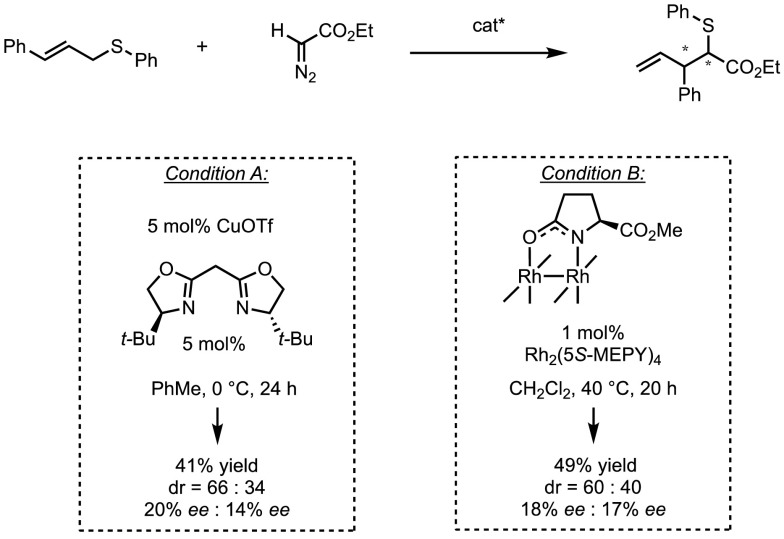
Enantioselective Doyle–Kirmse reaction by Uemura and co-workers

**Fig. 13:**
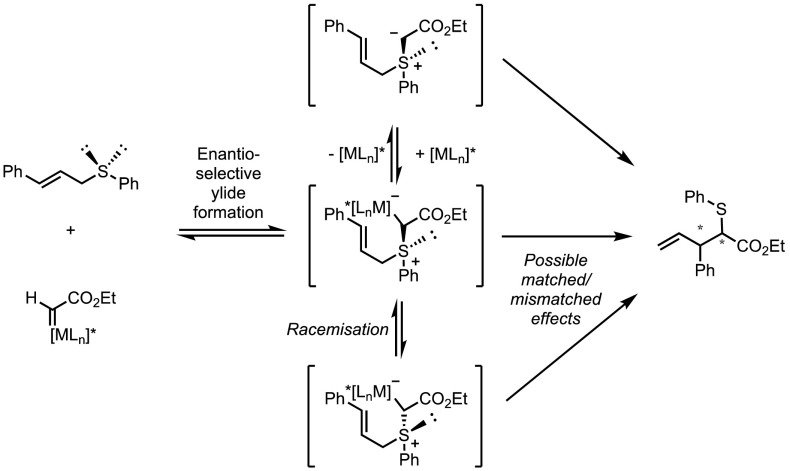
Plausible reaction pathways in enantioselective metal-catalysed Doyle–Kirmse reactions

The first enantioselective Doyle–Kirmse reaction was published by Uemura and coworkers in 1995 [96]. In the presence of Cu(I)/bisoxazoline or Rh_2_(5*S*-MEPY)_4_, the reaction between ethyldiazoacetate and (*E*)-cinnamylphenylsulfide resulted in the formation of chiral homoallylsulfides. However, only low levels of diastereo- and enantiocontrol could be obtained using these catalysts (Scheme 36). There are several explanations that could account for the low selectivity, and these are broadly summarised in Fig. 13. For example, the lack of discrimination between the two enantiotopic sulfide lone pairs during the initial attack onto the chiral metal carbene could be problematic. Furthermore, any stereocontrol at this step could be partially or totally negated by a fast racemisation process. In the absence of transition metal complexes, the configurational stability of sulfonium ylides is generally high, but whether this remains so in metal-catalysed rearrangements remains unclear.

**Scheme 37:**
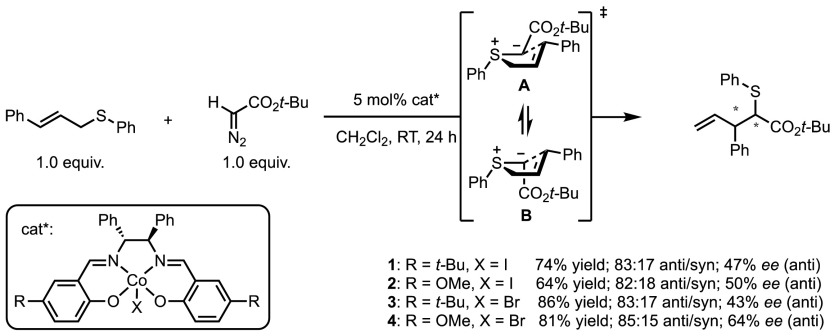
Cobalt salen-catalysed Doyle–Kirmse reaction

It is instructive that perfect chirality transfer was observed from [2,3]-sigmatropic rearrangements of enantiopure sulfonium ylides under metal-free conditions. However, the possibility of metal coordination could result in matched/mismatched effects. This has been studied by the group of Katsuki, who compared the influence of different Co-salen catalysts on the diastereoselectivity of their Doyle–Kirmse process (Scheme 37) [81]. While the enantiomeric ratios were found to be catalyst dependant, the diastereocontrol was the same for each catalyst. These authors argue that this observation suggests that while the sulfonium ylide formation is under catalyst-control, the rearrangement step occurs without catalyst coordination. The observed diastereoselectivity would therefore be a result of the potential energy difference between the two transition states **A** and **B**.

**Scheme 38:**
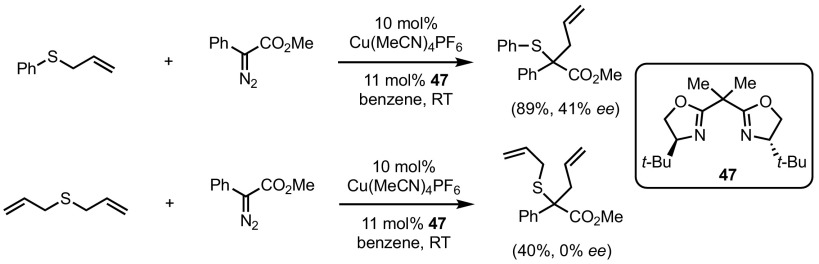
Difference in enantio-induction starting from non-symmetric and symmetric allylsulfides

Shortly thereafter, Hashimoto and co-workers [97] arrived at the same conclusions; however, Aggarwal and co-workers observed a strong correlation between ligand and diastereoselectivities [98]. In the Doyle–Kirmse reactions of allylsulfides and trimethylsilyl diazomethane, the latter authors found diastereoselectivities ranging from 1:1 to 9:1, but nearly racemic products. Further mechanistic studies undertaken by Wang and co-workers support a metal-free rearrangement step [99]. Although several rearranged sulfides with moderate enantioselectivities were synthesised with the combination of Cu(MeCN)_4_PF_6_ and bisoxazoline ligand **47**, no stereocontrol was observed when diallyl sulfide was used (Scheme 38).

**Fig. 14:**
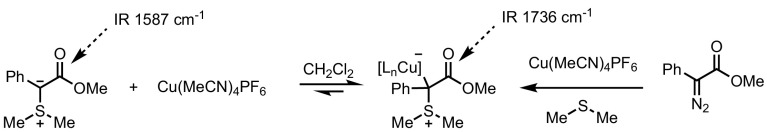
Evidence of the strong affinity of sulfonium ylides for electrophilic copper complexes

The ylide formed in this latter example is achiral and, therefore, a metal-free rearrangement would necessarily generate racemic product. On the other hand, the same authors were able to demonstrate a strong affinity between the copper catalyst and the sulfonium ylide by infrared spectroscopy studies (Fig. 14) [100]. The combination of these results suggests that the equilibrium favours the formation of the metal ylide complex, but that only the free ylide undergoes the rearrangement (or at least it does so much faster). Further studies by Trost and Hammen showed the final rearrangement step to be far faster than the racemisation of free sulfonium ylides under the reaction conditions [101]. Taking all these observations into account, the enantioselectivity of the whole sequence appears to be entirely dependent on the degree of enantiodiscrimination obtained in the first step, namely the formation of the chiral sulfonium ylide intermediate.

**Scheme 39:**
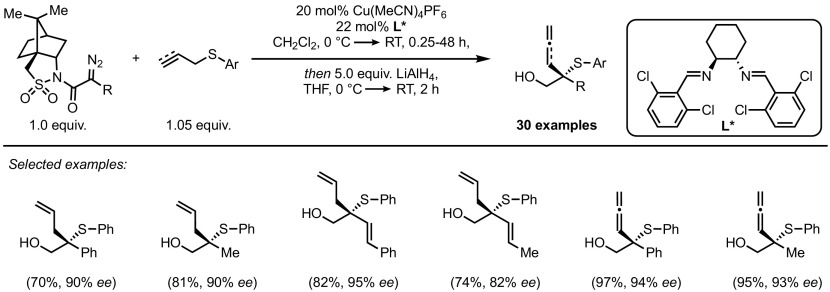
Double asymmetric induction in copper-catalysed Doyle–Kirmse reaction

For a long time, the best enantioselectivities were obtained through the use of double asymmetric induction, developed by Wang and co-workers [100]. Involving the combined actions of an enantiopure copper catalyst and Oppolzer’s chiral auxiliary, good to excellent enantiomeric ratios over a wide range of substrates could be achieved (Scheme 39). The reaction shows a broad scope of allyl- and propargylsulfides as well as tolerance of the use of aryl-, methyl-, cinnamoyl- and propenyldiazoacetamides. The auxiliary can be easily removed post-reaction though reduction. Nonetheless, the need for both a chiral catalyst and a chiral auxiliary suggests that this is not the ideal solution.

**Scheme 40:**
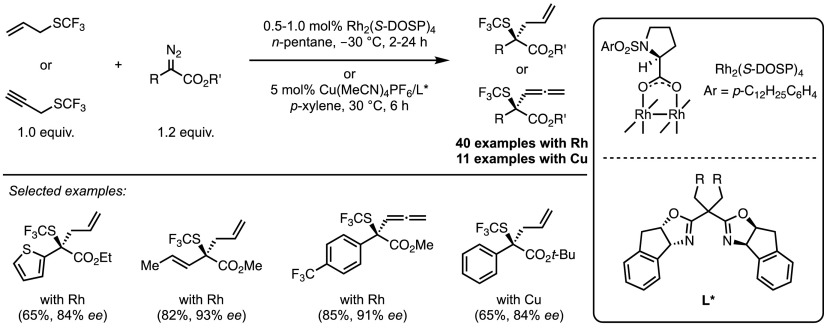
Enantioselective Doyle–Kirmse reaction under full catalyst control

Shortly before the publication of this review, the Wang group achieved similar levels of enantioselectivities without the need for a chiral auxiliary in the synthesis of enantioenriched trifluoromethylthio-substituted compounds (Scheme 40) [102]. The combination of a new set of ligands and highly electron-poor trifluoromethyl sulfides allowed for very high enantiocontrol over an unprecedented range of diazonium salts, including aryl-, alkenyl- and hetero-aryl- substituted diazoacetate derivatives. While enantioselectivities were high, low diastereoselectivities were observed when internal olefins were employed. Notably, these catalysts did result in non-racemic products when diallyl sulfide was employed (in contrast to the results depicted in Scheme 38), but the levels of enantiocontrol displayed were almost negligible. These observations support the previous proposals that decoordination of the chiral transition metal catalyst occurs prior to the [2,3]-rearrangement.

Diazo compounds are often excellent metal carbene precursors, as their decomposition in the presence of transition metal catalysts requires mild conditions and offers good chemoselectivity. However, they are associated with major drawbacks due to their hazardous and potentially explosive nature and are, consequently, of limited use for large-scale reactions. Furthermore, stable diazo compounds typically must carry at least one electron-withdrawing group. During recent decades, several alternative strategies have been laid forth that not only avoid the use of diazo compounds but also extend the scope and efficiency of the Doyle–Kirmse reaction. Promising results include the generation of diazo intermediates in situ and the direct generation of metal carbenoids from unsaturated C–C bonds in the presence of π-acid catalysts.

In this framework, tosylhydrazones are valuable precursors of unstabilised diazo compounds through the so-called Bamford–Stevens reaction. In the presence of catalytic Rh_2_(OAc)_4_ and allyl sulfides, tosylhydrazones were transformed into sulfonium ylides able to undergo [2,3]-rearrangement [103]. Compared to previously reported methods, this approach allows for the formation of rearranged products without the requirement for an electron-withdrawing group usually necessary to stabilise the diazo starting material. Another method for the in situ generation of diazo compounds is the slow addition of sodium nitrite to a primary amine, and such an approach was employed by Koenigs and co-workers [104]. Through the use of the appropriate amines, they were able to generate cyano-, ester- and trifluoromethyl-substituted diazo compounds which underwent Fe-catalysed Doyle–Kirmse reactions. While an improvement from the point of view of safety and scalability, this procedure does not yet offer as wide a scope as the Bamford–Stevens-based chemistry.

**Scheme 41:**
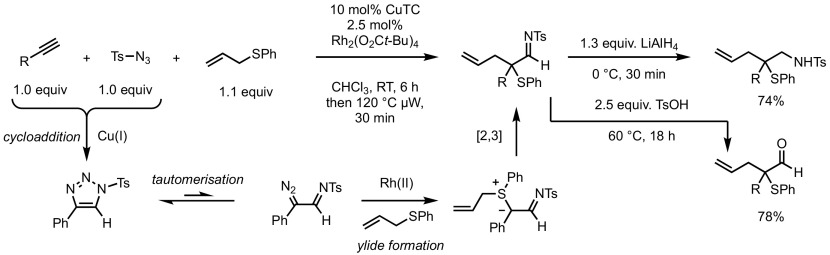
In situ generation of diazo compounds in the Doyle–Kirmse synthesis of thioether imines

More recently, the use of 1-sulfonyl-1,2,3-triazoles as masked diazoimines was also exploited. Murakami and co-workers were the first to report this strategy in combination with the [2,3]-rearrangement of allylsulfonium ylides [105]. They propose a cascade reaction starting with a copper-catalysed (3 + 2)-cycloaddition from readily available alkynes and tosylazides leading to triazole intermediates (Scheme 41). The diazoimine tautomers of these triazoles are then decomposed in the presence of the second catalyst Rh_2_(O_2_C*t*-Bu)_4_ to generate the metal-carbene complex. Attack of the allylsulfide, followed by [2,3]-sigmatropic rearrangement afforded the desired sulfenylated imines that can be either hydrolysed to the corresponding aldehyde or reduced with LiAlH_4_ to give tosylamines. The yields are surprisingly high and demonstrate a remarkable chemoselectivity for such a complex sequence. Shortly thereafter, a similar strategy for rhodium-catalysed denitrogenative synthesis of α-sulfenylated imines was reported by Yadagiri and Anbarasan [106].

**Scheme 42:**
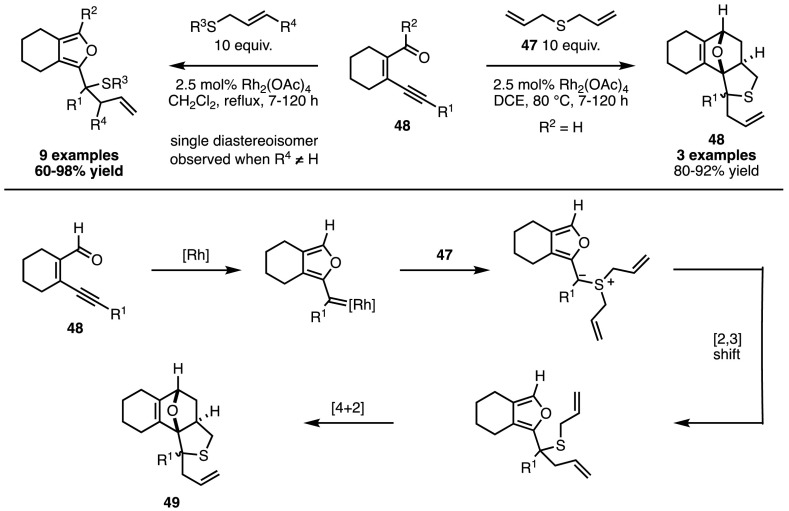
Doyle–Kirmse reactions from in situ-generated (2-furyl)carbenoids

While the in situ generation of diazo compounds represents an improvement from the perspective of safety and operational simplicity, methods that can generate metal carbenoids without the need for extrusion of nitrogen gas are a further improvement. In 2002, Ohe and Uemura developed one such method, in which they used rhodium diacetate to promote the cycloisomerisation of ene-yne-carbonyl compounds (**48**) to rhodium-bound (2-furyl)carbenoids [107]. By combining these metal-carbenoids with allyl sulfides, it was possible to access both the products of the Doyle–Kirmse reaction in good to excellent yields, and, in cases where diallyl sulfide **47** was employed, the polycyclic products (**49**) of a further [4 + 2] cycloaddition (Scheme 42) [108].

**Scheme 43:**
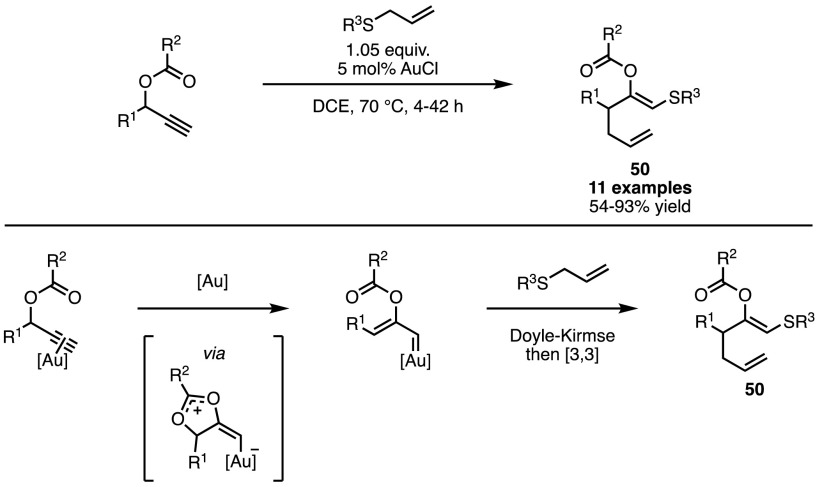
Alkynes as masked ylides in Doyle–Kirmse chemistry

Among other potential carbenoid precursors are propargyl carbonates. Through migration of the acyl unit, highly reactive, conjugated metal-carbenoids can be generated. In 2008, Davies and Albrecht used this approach to form products of the Doyle–Kirmse reaction using AuCl; however what these authors observed was that instead of the expected homo-allyl sulfides, the isolated product was structure **50** (Scheme 43) [109]. The precise mechanism of this transformation remains unclear, but the rearrangement of the expected Doyle–Kirmse products to **50** via a subsequent [3,3] sigmatropic shift would provide a plausible explanation.

**Scheme 44:**
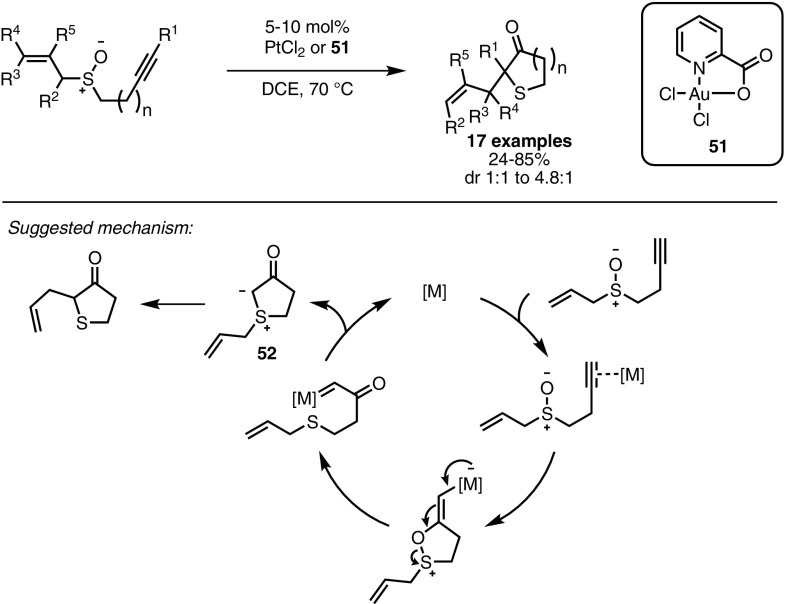
Synthesis of sulfur heterocycles from alkynyl sulfoxides

In 2009, the same authors proposed a similar strategy for the synthesis of cyclic sulfides [110]. Based on the works of Shapiro and Toste [111] and Li and Zhang [112], alkyne oxidation was achieved through the intramolecular addition of a sulfoxide moiety, promoted by the π-acidic catalyst (Scheme 44). Following cleavage of the S–O bond, the resulting metal carbene and sulfide collapsed intramolecularly to form ylide **52**. A final [2,3]-rearrangement afforded heterocyclic products. Optimisation of the reaction conditions revealed PtCl_2_ to be the best choice of catalyst for the reaction with terminal alkynes, whereas for internal alkynes, the use of dichloro(pyridine-2-carboxylato)gold(III) **51** as a catalyst was required for the efficient synthesis of cyclic sulfides bearing a quaternary stereocentre. Ester- and aryl-substituted internal alkynes were well tolerated, while highly substituted allyl sulfoxides afforded the desired product, but only in low diastereoselectivity.

More recent methodologies concerning the intermolecular oxidation of alkynes using stoichiometric amounts of amine *N*-oxides have also been reported. These procedures have the advantage of not having to use highly designed substrates, but rather they can use simple starting materials. As the potential for the formation of regioisomeric mixtures is more of a problem in intermolecular processes, polarised alkynes (e.g. ynamides) were chosen for the oxidative synthesis of 2-(thio)amides bearing a α-quaternary centre [113]. Only a narrow scope of ynamides was presented, which suggests difficulties in controlling the chemoselectivity in the presence of alternative external nucleophiles that can intercept the gold carbene intermediate. This competition was addressed in an independent publication by Zhang and co-workers describing the three-component synthesis of α-aryl(alkyl)thio-γ,δ-unsaturated ketones starting from terminal alkynes [114]. This reaction could be carried out efficiently by the addition of the oxidising reagent via a syringe pump and, more importantly, *P*,*S*-bidentate ligands were used to decrease the electrophilicity of the α-oxo gold carbene complex.

**Scheme 45:**
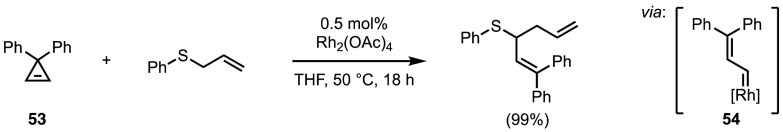
Doyle–Kirmse reaction from cyclopropenes

The most recent developments for the diazo-free Doyle–Kirmse reaction relied on the cyclic strain of cyclopropenes [115]. Treatment of cyclopropene **53** with catalytic Rh_2_(OAc)_4_ resulted in spontaneous ring opening to form the corresponding rhodium carbene **54** (Scheme 45). This intermediate could react with allyl(phenyl)sulfide to afford a sulfonium ylide that immediately undergoes rearrangement. This approach was quite general, allowing the synthesis of sulfur-containing alkenes and allenes in good yields. Attempts to deliver an asymmetric version only resulted in low enantioselectivities.

### 1,2-Migration of Sulfonium Ylides

While being powerful transformations, the Doyle–Kirmse and Sommelet–Hauser reactions by no means represent the only rearrangements available to sulfur-based ylides. Direct 1,2-migrations of alkyl substituents from the cationic to the anionic centre are known as Stevens rearrangements. These reactions have been thoroughly investigated in systems based on oxonium and ammonium ylides, but the sulfur-equivalent, i.e. thia-Stevens rearrangement, has not received nearly as much attention. Though underutilised, the thia-Stevens rearrangement has still proven to be a powerful tool for C–C (and C–N [116]) bond formation and the synthesis of quaternary centres.

When combined with the power of in situ, transition metal-mediated ylide formation, the synthetic potential of this synthetic strategy is fully unlocked. In particular, such processes have most commonly been used for the synthesis of sulfur-containing heterocycles through ring-expanding 1,2-shifts.

**Scheme 46:**
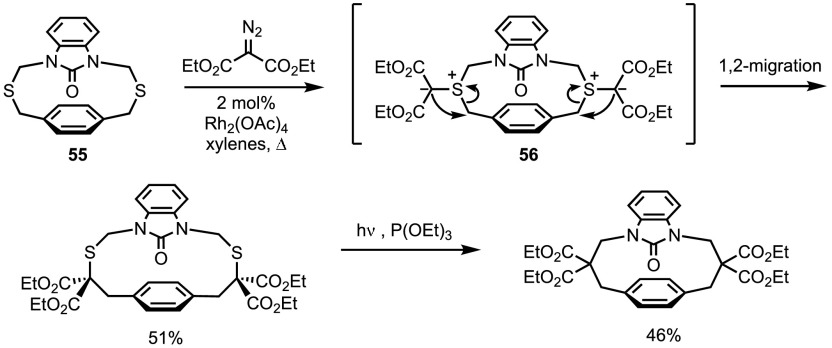
Macrocycle ring expansion by the double Stevens rearrangement

One such report involves the ring-expansion of sulfur-containing macrocycles via a double thia-Stevens rearrangement (Scheme 46) [117, 118]. Reported by the group of Diver, 2 mol% of rhodium acetate is sufficient to promote the formation of ylide **56** from the combination of disulfide **55** and diethyl diazomalonate. Although this ylide can be isolated at low temperatures, performing the reaction in refluxing xylene allows the 1,2-sigmatropic shift to readily occur, affording the ring-expanded macrocyclic product in 51% yield. The authors state that the regioselectivity of the migration step suggests a radical mechanism. When exposed to triethyl phosphite under photolytic conditions, sulfur extrusion can be triggered to afford [3,3]-heterophanes.

**Scheme 47:**
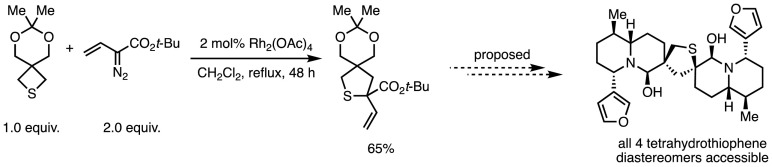
Proposed synthesis of *Nuphar* thioalkaloids through ring expansion of thietanes

Smaller rings, such as tetrahydrothiophenes, can also be synthesised in this manner [117], a process that has been applied in the synthesis of complex structures akin to the *Nuphar* thioalkaloids thiolane core (Scheme 47) [119].

**Scheme 48:**
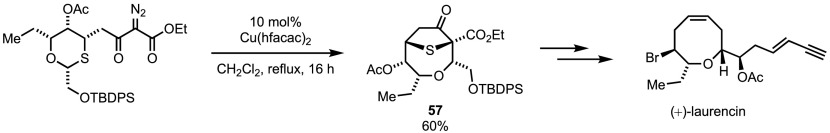
Stevens rearrangement as a key step in the formal synthesis of (+)-Laurencin

Further application to natural product synthesis can be found in a report by West and co-workers, who used the 1,2-migration of a monothioacetal-derived ylide as a key step in the formal synthesis of (+)-Laurencin [120]. Medium-sized thioether **57** was thus obtained in a 60% yield (Scheme 48). Intriguingly, the α-stereogenic centre was perfectly conserved during the 1,2-migration. If such Stevens rearrangements indeed proceed with high levels of stereochemical fidelity, an enantiocontrolled version would be highly attractive and synthetically useful.

**Scheme 49:**
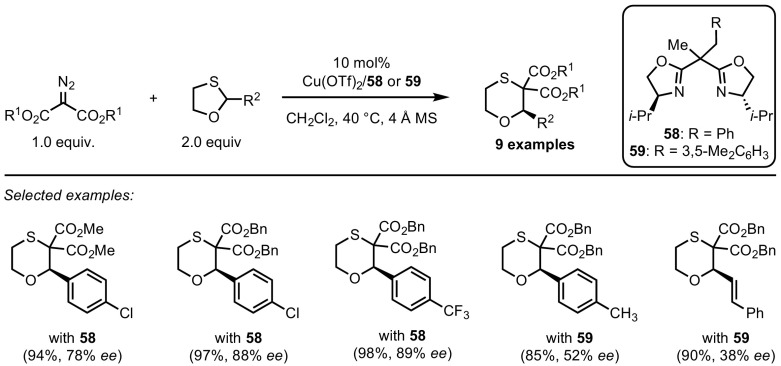
Copper-catalysed enantioselective 1,2-migration of dithioacetals

Such a process was reported in 2009 by Tang and co-workers [121]. Through the action of the complex formed from copper(II) triflate and chiral bisoxazoline **58**, they were able to synthesise a number of six-membered 1,4-oxathianes from diazomalonates and racemic 1,3-oxathiolanes in an ylide formation/Stevens rearrangement domino sequence (Scheme 49). Yields were mostly good, and the enantioselectivities promising; more electron-rich substrates required the use of ligand **59**, which, while more reactive, offered reduced enantiocontrol. In this system, sulfonium ylides with allylic functionality, demonstrated in Sect. 5.2 to be good substrates for 2,3-sigmatropic shifts, exclusively underwent the 1,2-shift.

**Scheme 50:**
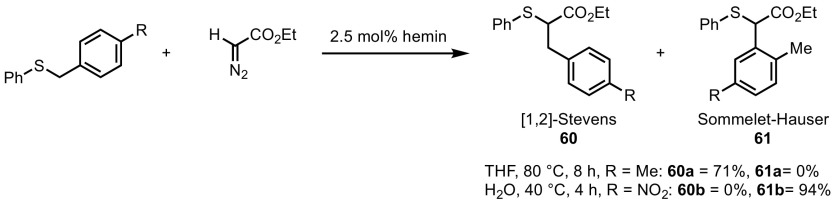
Chemoselectivity between 1,2-migration and Sommelet–Hauser rearrangement

During competition studies undertaken by Pan and co-workers to further probe this selectivity, it was shown that the reaction outcome strongly depends on the reaction conditions and electronic properties of the substrates [122]. The hemin-catalysed formation and rearrangement of benzyl sulfonium ylides led to outcomes dependant on both the nature of the solvent and the presence/absence of electron-withdrawing substituents on the aromatic ring (Scheme 50). The authors established that, in protic solvents and with electron-poor benzylic substituents, the [2,3]-Sommelet–Hauser rearrangement (leading to **60**) is the dominant pathway, whereas with electron-rich substrates, only the Stevens rearrangement (leading to **61**) was observed.

### Synthesis of Small Rings

Beyond rearrangement reactions, any number of other ylide-mediated transformations can be achieved through metal-catalysed, in situ generation of the sulfonium ylide, rather than using a preformed reagent. The prototypical example of such chemistry is the synthesis of small rings through the Corey–Chaykovsky reaction. These processes have long been studied, with highly diastereo- and enantioselective reactions reported. The reader is referred to several in-depth reviews and book chapters on this topic for further information [123, 124].

#### Epoxidation

The synthesis of epoxides as synthetic intermediates is a rich field in synthetic methodology. Whilst a wide array of catalytic alkene oxidation conditions have been developed, there are often advantages to using alternative disconnections in which enantioinduction can be more easily achieved. The synthesis of enantiopure epoxides is of key importance, as the stereoselective ring opening of such structures with suitable nucleophiles can create contiguous chiral centres. Chiral variants of the Corey–Chaykovsky reaction are well precedented, although the majority of these reactions are stoichiometric in the chiral sulfonium. Catalytic variants are possible, although the scope of these transformations remains limited.

**Scheme 51:**
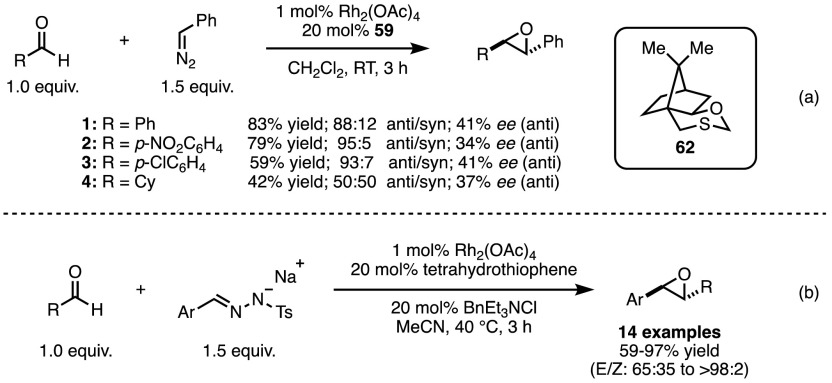
Diazo-based and diazo-free sulfide-mediated epoxidation of aldehydes

Aggarwal and co-workers demonstrated in 1994 that the concept of transition metal-promoted sulfonium ylide synthesis could be used to generate transient chiral ylides. These ylides were then able to effect epoxidation, with the expulsion of the chiral sulfide, which would go on to react with a further metal carbenoid to propagate the cycle [125]. Catalytic in both the sulfide and transition metal, the process relied on the slow addition of the diazo compound in addition to high dilution conditions. Yields were significantly reduced without the combination of these strategies. As metal catalysts, both Cu(acac)_2_ and Rh_2_(OAc)_4_ were effective at promoting the formation of the sulfonium ylide intermediate. Further studies investigated the use of chiral sulfides such as **62** (Scheme 51a) [126–128].

**Fig. 15:**
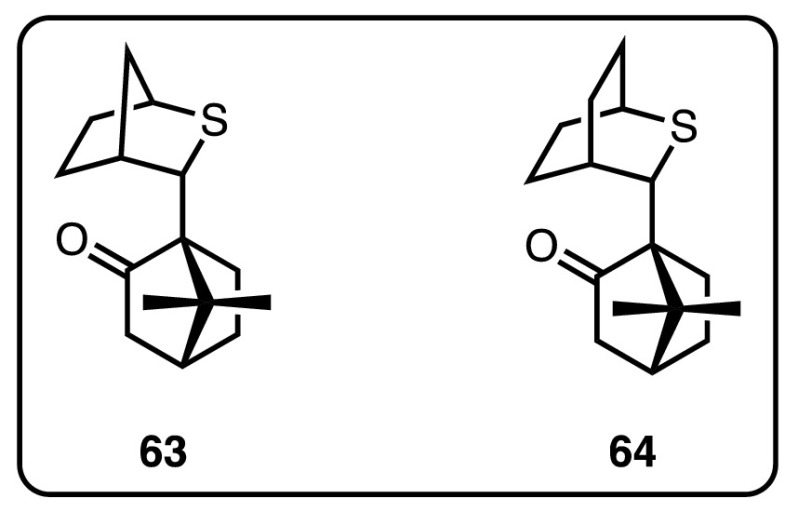
Camphor-derived chiral sulfides for epoxidation, aziridination and cyclopropanation

In order to overcome the necessity of employing large quantities of potentially hazardous (and often structurally limited) diazo compounds, a modified procedure was developed in which tosylhydrazones could be used as safer surrogates of diazo compounds (Scheme 51b) [129, 130]. Such easily accessible tosylhydrazone salts were decomposed in acetonitrile under mild reaction conditions, with phase transfer catalysts used to provide a slow release of diazo equivalent into solution. Camphor-derived sulfide **63** (Fig. 15) could be used to generate chiral epoxides, with moderate to good yields and high enantio- and diastereocontrol, in particular with electron-rich aryl tosylhydrazone salts. Alternative substrates including electron-poor aryl and other *sp*^2^-substituted substrates experienced varying degrees of success.

#### Aziridination

By exchanging the aldehyde component for an imine, it is possible to access aziridines. These structures are synthetically useful building blocks for similar reasons to epoxides, and a number of asymmetric methods for their construction have been developed. Aggarwal demonstrated an extension to his above-mentioned epoxide synthesis in 1996 by utilising electron-poor imines, affording protected aziridines [131]. The necessity of the electron-withdrawing functionality on the nitrogen was a result of the ability of metal carbenoids to react with imines directly, negating the effect of the chiral sulfide. The higher electrophilicity of the electron-poor imines renders them at once more reactive towards the sulfonium ylide and less prone to react with the metal carbenoid. In addition to diazo compounds, tosylhydrazone salts were again suitable carbenoid precursors [132, 133], and ketimines and alkyl aldimines were successfully employed in the process with no loss of reaction efficiency or yield.

#### Cyclopropanation

Aggarwal and co-workers further demonstrated that the strategy of in situ generation of sulfonium ylides was applicable to the synthesis of cyclopropanes from electron-deficient olefins [134]. While the enantioselectivities of the generated cyclopropanes were excellent, the yields remained moderate while using sulfide **63**, previously employed in epoxidations and aziridinations. Higher yields of the cyclopropane products were obtained by switching to sulfide **64** (Fig. 15) [31]. Finally, it was once again shown that the use of tosylhydrazone salts in lieu of diazo compounds could be achieved [132].

### In Situ Generation of Thiocarbonyl Ylides

**Scheme 52:**
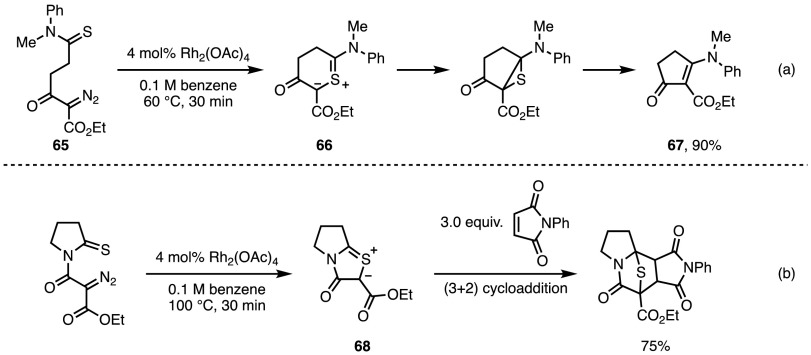
Rhodium-catalysed formation of thiocarbonyl ylides for electrocyclisation and cycloaddition reactions

Thiocarbonyl ylides, formally 1,3-dipoles, represent a conceptually distinct class of sulfonium ylides with unique reactivity [135]. High-energy compounds, these ylides can react via electrocyclisation, dimerisation-type reactions, 1,3 acid–base addition reactions, rearrangements and cycloadditions. While normally generated through alkylation/deprotonation or through nitrogen extrusion from thiadiazolines, a number of publications on the use of in situ-generated thiocarbonyl ylides can be found. The first reports of this technique involved the combination of malonate-derived diazo compounds with di-*tert*-butyl thioketene, and a number of rearrangement products were observed [136, 137]. Of more synthetic utility was a process reported by Padwa et al. [138], in which a 3-amino cyclopentanone (**65**) could be generated from a thioamide-tethered diazo compound **67**, via the intermediacy of a cyclic thiocarbonyl ylide **66** (Scheme 52a) [138]. In this process ylide **66** undergoes a Woodward–Hoffmann-forbidden 1,3-electrocyclisation to generate a thiirane, which in turn extrudes elemental sulfur to form the desired five-membered ring. A similar, intermolecular version of this olefination reaction was employed by Danishefsky and co-workers in the synthesis of Indolizomycin [139, 140].

Further experiments by Padwa and co-workers on similar substrates found that if the cyclic thiocarbonyl ylide had additional stabilisation, such as in the mesoionic, aromatic thiaisomünchnone **68**, then the compound is stable and isolable or, alternatively, it can react in situ with suitable dipolarophiles in a (3 + 2) fashion (Scheme 52b) [141]. Ylide **66**, even in the presence of an excess of dipolarophile was not observed to undergo this cycloaddition, with the (1,3) electrocyclisation occurring much more rapidly.

## Outlook

Throughout each of the sections in this review, we have demonstrated how the power and flexibility of transition metal catalysis can be harnessed for the development of innovative, novel transformations involving sulfonium and sulfoxonium ylides. Such ylides traditionally react as nucleophiles, which restricts their application as single carbon synthons to a handful of valuable, but limited reactions. By introducing transition metal catalysts into the equation, a considerable breadth of reactivity can be accessed. A number of enantioselective transformations have been demonstrated utilising chiral ligands, often through the enantiocontrolled, in situ generation of chiral sulfonium ylides for use in cascade processes. Whether through carbene transfer, oxidative addition pathways or Lewis acid activation, the use of transition metal catalysts in combination with sulfur ylides represents an underexplored and underutilised area of synthetic organic chemistry, where further exciting developments can be anticipated in coming years.

## Abbreviations

Δ: Heat
Ac: Acetyl
Ar: Aryl
BINAP: 2,2'-Bis(diphenylphosphino)-1,1'-binaphthyl
COD: Cyclooctadiene
Cy: Cyclohexyl
dba: Dibenzylideneacetone
DCE: 1,2-dichloroethane
DFT: Density functional theory
DMA: Dimethylacetamide
DMF: Dimethylformamide
DMSO: Dimethylsulfoxide
dr: Diastereomeric Ratio
*ee*: Enantiometric excess
GCMS: Gas Chromatography–Mass Spectrometry
hfacac: Hexafluoroacetylacetone
HFIP: Hexafluoroisopropanol
hv: Light
IMes: 1,3-Dimesitylimidazol-2-ylidene
IR: Infrared
LED: Light emitting diode
MS: Molecular sieves
NMR: Nuclear magnetic resonance
*p*-TSA: *p*-Toluenesulfonic acid
RT: room temperature
SET: Single electron transfer
TC: Thiophene 2-carboxylate
Tf: Trifluoromethanesulfonyl
TFA: Trifluoroacetic acid
THF: Tetrahydrofuran
Tol: Tolyl, 4-methylphenyl
Ts: Tosyl, *p*-toluenesulfonyl
UV: Ultraviolet
μW: Microwave heating
